# Proactively averting the collapse of Amazon fisheries based on three migratory flagship species

**DOI:** 10.1371/journal.pone.0264490

**Published:** 2022-03-02

**Authors:** Luiza Prestes, Ronaldo Barthem, Adauto Mello-Filho, Elizabeth Anderson, Sandra B. Correa, Thiago Belisario D’Araujo Couto, Eduardo Venticinque, Bruce Forsberg, Carlos Cañas, Bianca Bentes, Michael Goulding

**Affiliations:** 1 Programa de Graduação em Engenharia de Pesca, Universidade do Estado do Amapá, Macapá, Amapá, Brazil; 2 Museu Paraense Emílio Goeldi-Campus de Pesquisa-Coordenação de Ciências da Terra e Ecologia, Belém, Pará, Brazil; 3 Programa de Pós-Graduação em Ecologia Aquática e Pesca, Universidade Federal do Pará, Belém, Pará, Brazil; 4 Florida International University, Institute of Environment, Miami, Florida, United States of America; 5 Aquatic Ecology, Department of Wildlife, Fisheries and Aquaculture, Mississippi State University, Starville, Mississippi, United States of America; 6 Universidade Federal do Rio Grande do Norte (UFRN), Natal, Brazil; 7 Vermont Department of Environmental Conservation, Montpelier, Vermont, United States of America; 8 Battelle, National Ecological Observatory Network, Gainesville, Florida, United States of America; 9 Laboratório de Biologia Pesqueira e Manejo de Recursos Aquáticos, Núcleo de Ecologia Aquática e Pesca, Universidade Federal do Pará, Belém, Pará, Brazil; 10 Wildlife Conservation Society (WCS), New York, NY, United States of America; DePaul University, UNITED STATES

## Abstract

Migratory species are the most important commercial fishes in the Amazon. They are also now the most threatened directly by some combination of overfishing, floodplain deforestation, and dam construction. Limited governmental monitoring and implemented regulations impede adequate management of the fisheries at adequate scale. We summarize the current stock status of the three most heavily exploited long-distance migratory species, which are two goliath catfishes (*Brachyplatystoma rousseauxii* and *B*. *vaillantii*) and the characiform *Colossoma macropomum*. In addition, we analyze impacts beyond overfishing on these species. Our results indicate: (i) the overfishing trends for these important species are either ominous or indicate the verge of collapse of the commercial fisheries based on them, and (ii) a dangerous synergy between overfishing, hydroelectric dams, and floodplain deforestation further challenge fisheries management of migratory species in the Amazon. We propose eight direct governmental actions as a proactive approach that addresses the main impacts on the fisheries. We consider that the most practical way to assess and manage overfishing of migratory species in the short run in an area as large as the main commercial fishing area in the Amazon is at market sites where enforced regulations can control fish catch. The management of the three species considered here has implications beyond just their sustainability. Their management would represent a paradigm shift where the governments assume their legal responsibilities in fishery management. These responsibilities include regulation enforcement, data collecting, inter-jurisdictional cooperation to protect migratory species at realistic life history scales, mitigation of the Madeira dams to assure goliath catfish passage to the largest western headwater region, and recognition of monitoring and managing wetland deforestation for the protection of fish and other aquatic and terrestrial biodiversity.

## Introduction

**Fig 1:**
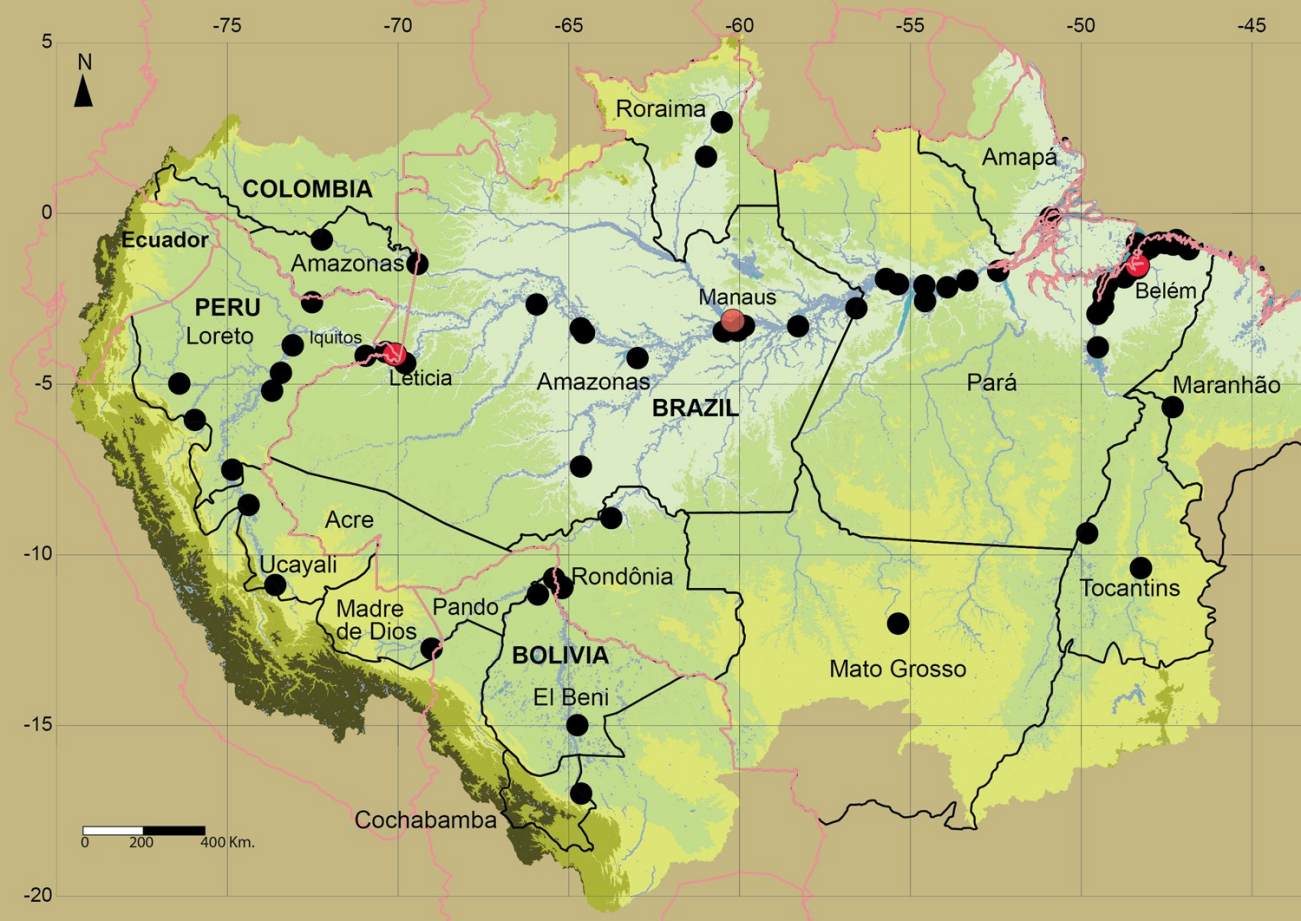
Area and fishery production of long-distance migratory catfishes. The Amazon region, highlighting the countries, departments (Bolivia, Peru, Colombia) or states (Brazil), and fish markets (black dots) where data have been collected, and the main fish landing cities (red points).

Migratory species are the most important fishes captured in the commercial fisheries of the Amazon Basin [1] (Fig 1). This is due to their abundance, size, schooling behavior, relatively high market prices, and culinary appreciation [2–7]. Stock assessment and precipitous declines in annual captures indicate the collapse of several important migratory fish species in the Amazon that are important in the ecology of Amazon waters, to fisheries, and as cultural icons. The management of migratory fish species in the Amazon faces major challenges. There is no legal recognition of which species are considered migratory and in need of special protection, how many stocks (isolated populations) exist of each species, and the critical life history area of each species. Those management strategies that have been implemented, which in most cases are led by academics and NGOs, have been focused on local problems that are insufficient to address the scale of fish migrations. Basin-wide management is further hindered by the diverse political geography of the Amazon Basin, non-continuous fish market data collection, ambiguous fisheries policies, and especially lack of government regulations and interventions to avert overfishing and aquatic ecosystem degradation [8–12].

The management of long-distance migratory fish species can only be addressed effectively and realistically at their life history scales by governmental integrated and centralized actions. These actions include monitoring and assessment of fish stocks, the use of science-based knowledge to develop rules and implement fisheries regulations, the mitigation of infrastructure impacts on fisheries, and the conservation of wetlands on which fish depend [13–15]. Considering these challenges, we propose that immediate steps should begin with basic actions that are realistically implementable, such as the management of migratory species that are on the verge of collapse due to overfishing. Our working assumption is that if overfishing of migratory species is not controlled, then there will be little incentive to protect other less harvested migratory species and their wetland habitats, and thus the fisheries in general. In this scenario, the elimination of the long-distance migratory species would represent a tipping point not only for Amazon fisheries, but also for aquatic biodiversity in general due to a failure to address realistic management scales.

**Fig 2:**
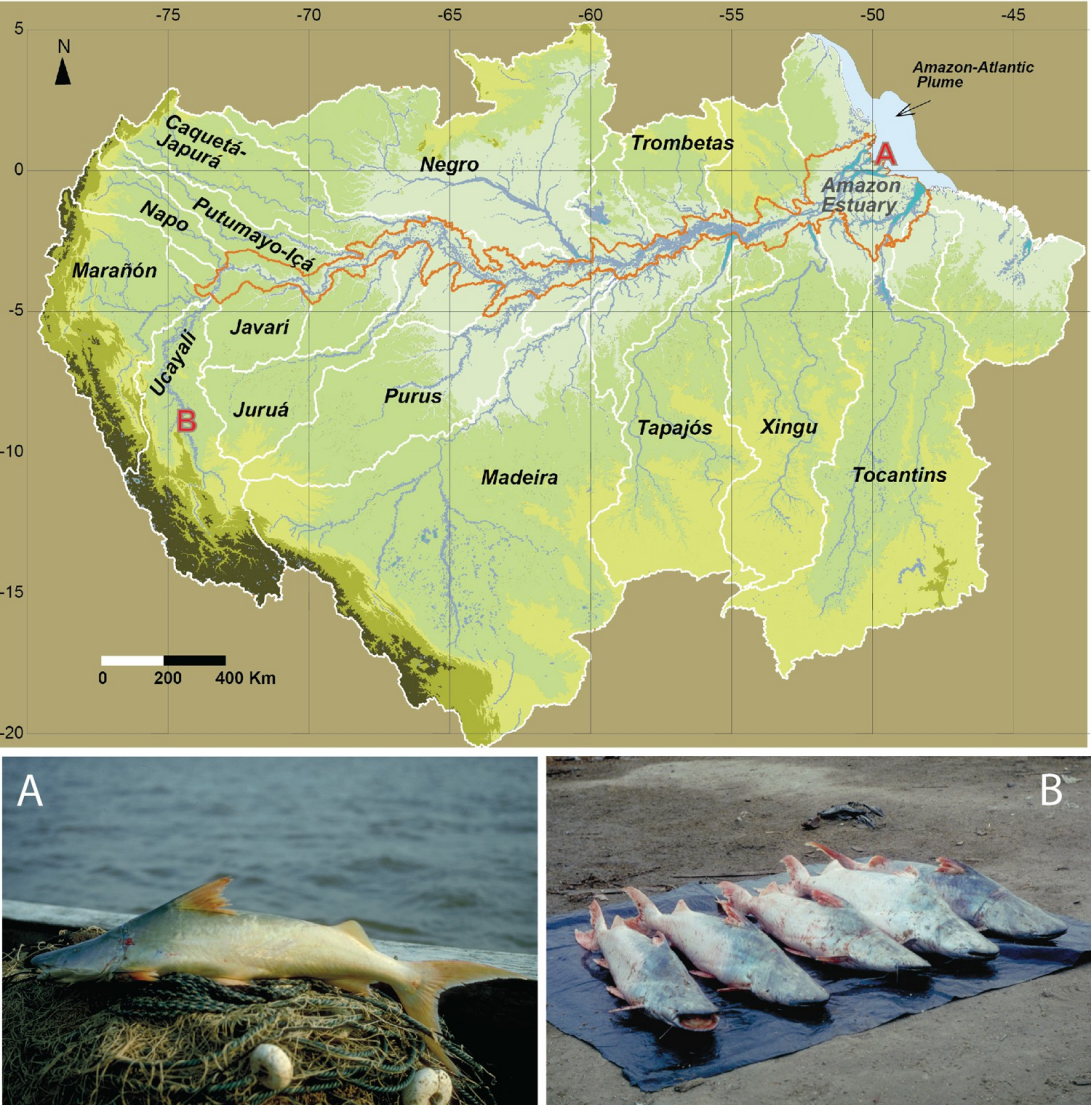
Fishing *B*. *rousseauxii* across the Amazon Basin. (A) A young *B*. *rousseauxii* captured in the eastern Amazon River at about the size (50 cm fork length) when the species first begins to migrate upstream in the Amazon River; (B) adult fish in the western Amazon near the Andes that were ready to spawn.

**Fig 3:**
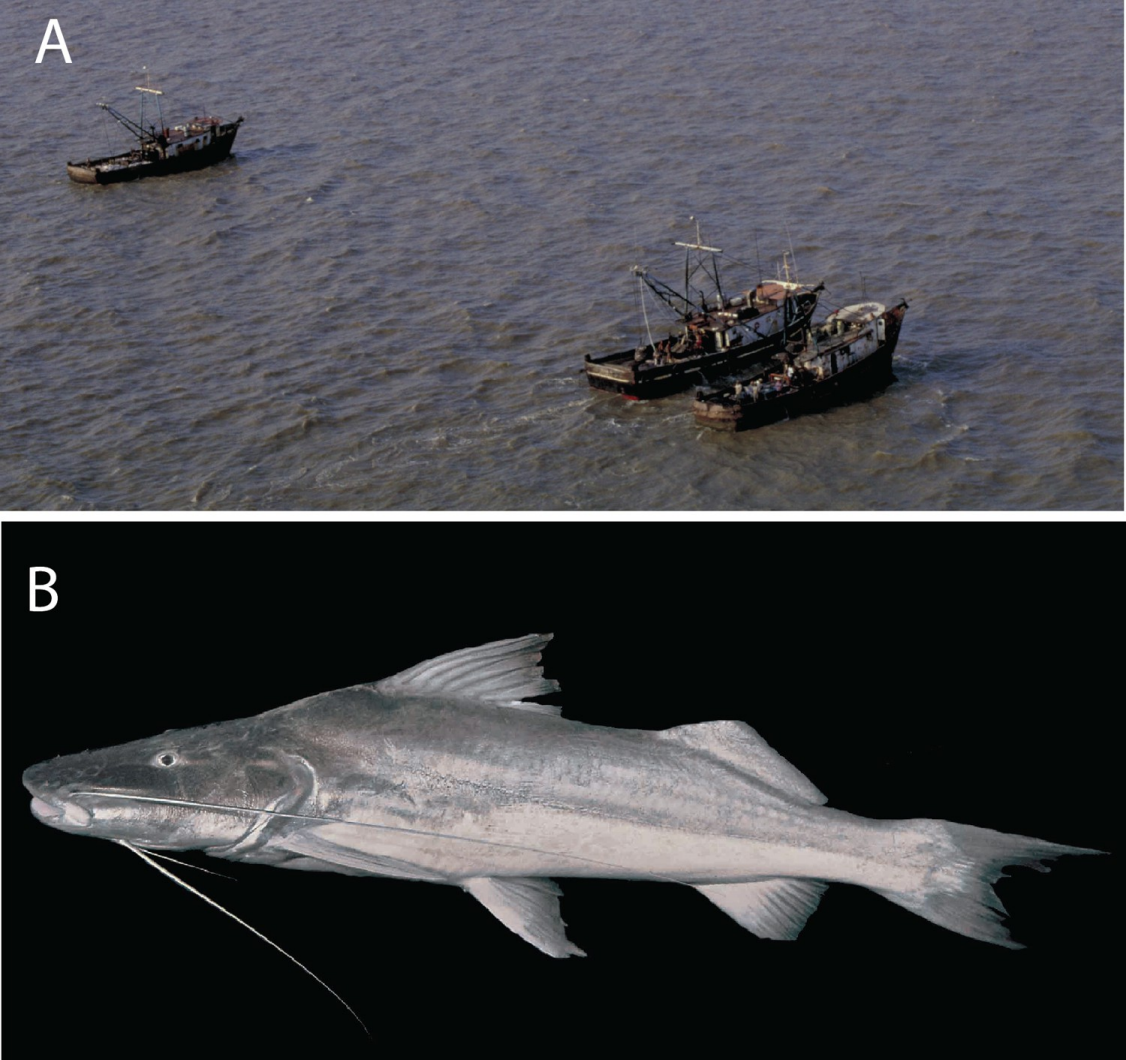
Industrial trawl fishery for *B*. *vaillantii*. Trawl fisheries in the open waters of the Amazon estuary adjacent to the mouth of the Amazon River account for most of the *B*. *vaillantii* catch.

**Fig 4:**
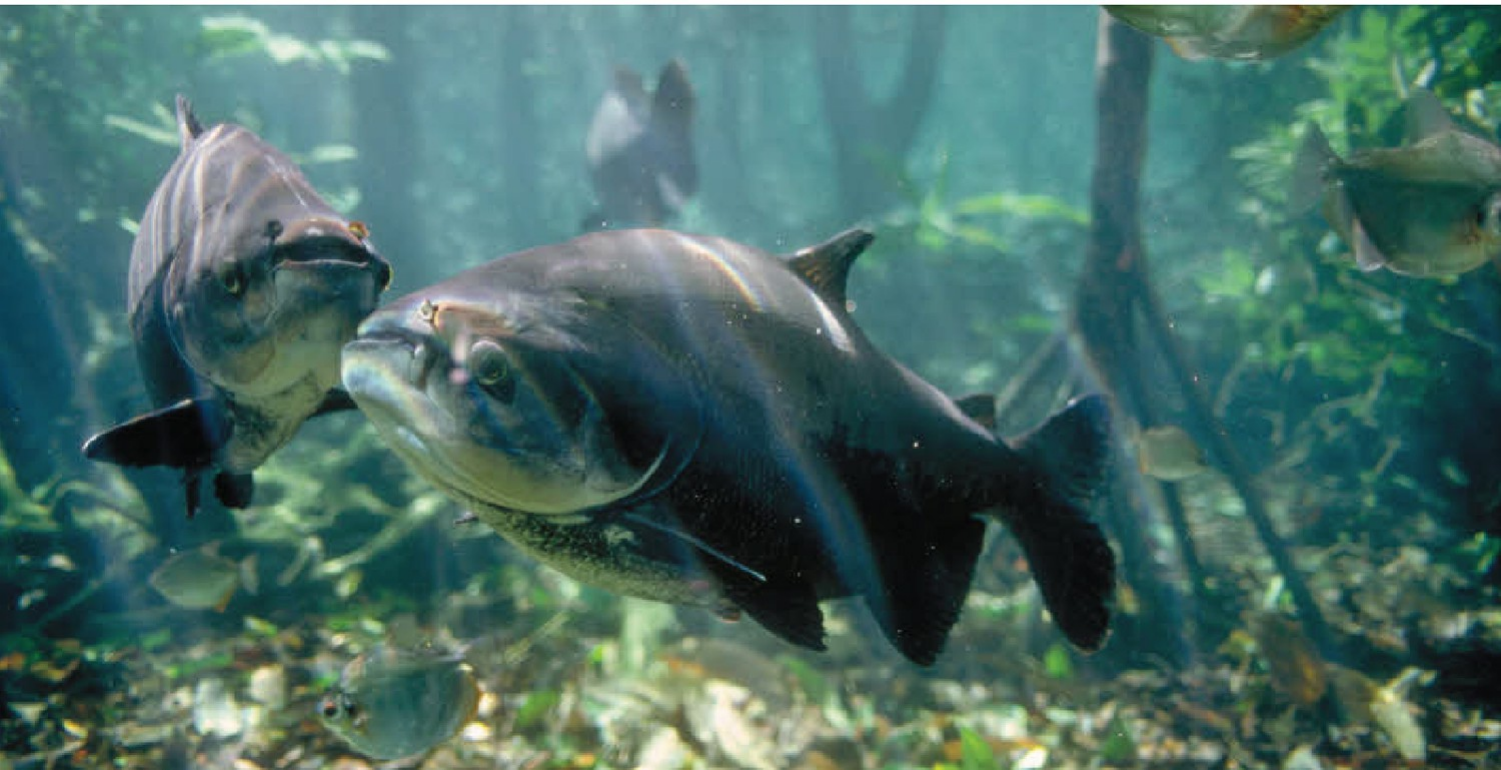
Colossoma *macropomum* in the Central Amazon. Adult C. *macropomum* are now rare in fisheries because of decades of overexploitation of both mature and immature fish. Floodplain deforestation along the lower Amazon River has also impacted the species.

We focus on overfishing of the most overexploited migratory species, which are the goliath catfishes, *Brachyplatystoma rousseauxii* (Fig 2) and *B*. *vaillantii* (Fig 3), and the characiform *Colossoma macropomum* (Fig 4) (Table 1). All have been or currently are among the most important commercial fishes in the Amazon. Although many commercial species are exploited inland in a manner typical for multi-species, multi-gear operations, few are as highly targeted as the goliath catfishes, and none more so than *C*. *macropomum* [1, 16–19]. Their overexploitation and stock status in Amazon fisheries makes them ideal for addressing the scale at which fisheries based on migratory fishes need to be managed [8, 14].

**Table 1:** Scientific and common names of three long-distance migratory species.

Scientific Name	Common Names
*Brachyplatystoma rousseauxii* (Castelnau, 1855)	EN: Gilded catfish; PO: Dourada; SP: Dorado, Plateado
*Brachyplatystoma vaillantii* (Valenciennes, 1840)	EN: Laulao catfish; PO: Piramutaba; SP: Pirabutón, Manitoa
*Colossoma macropomum* (Cuvier, 1816)	EN: Blackfin pacu; PO: Tambaqui-zebra; SP: Gamitana, Pacu, Cachama-negra

EN: English; PO: Portuguese; SP: Spanish

## Methods

### Study area

This study focuses on the main commercial fishing area in the Amazon Basin and the life history areas of the three most overexploited migratory species. The commercial fishing area is defined here as that which is fished for urban fish markets between the Andean Piedmont and the freshwater areas off the Amazon estuary in the east (Fig 1). The life history areas of the migratory species considered here are based on those already defined [8, 14].

### Data sources

Fisheries data included annual fishery catches from different sources between 1976 and 2019, though with incomplete information for all years and geopolitical units. These data were used as an indication of the potential importance of each species for each geopolitical unit to estimate the Maximum Sustainable Yield (MSY) of the industrial fishery in the estuary, and to project the fishery trends for the other species. Fish length data from goliath catfish fisheries in the estuary and in the Andean piedmont were used to assess stock health. Further details on the fisheries data sources are provided in S1 Text.

### Data analyses

The complex assemblage of data was analyzed with the objective to determine: (i) the importance of each geopolitical unit in the total production of the studied species, (ii) the stock status of the goliath catfishes exploited by the industrial fishery in the estuary and by the artisanal commercial fishery in the Andean piedmont, and (iii) for *B*. *rousseauxii* and *C*. *macropomum* the time series analyses of the fish stock status based on historical catches.

#### Annual fish catch analyzes

Long-term data obtained from different sources generated the time series of the annual landings by department, state, and urban centers. Due to the limitation of the available data and the uncertainty of the data collection methodology used in different countries, states, departments, and cities, we consider the maximum annual catch per species as indicative of their potential importance in the fisheries, though not indicative of sustainable yields. Time trends in fish landings were estimated for urban centers with high production of the analyzed species and significance was inferred with linear regression.

#### Fishery stock assessment

The status of goliath fish stocks was determined by the trends of two biological indicators: (1) the relationship between the current fishing mortality (F), and the F that produces Maximum Sustainable Yield (MSY) (F/F_MSY_) [20, 21], estimated for both species and all regions, and (2) the relationship between the present catch and the MSY obtained through the relative rate of catch increase (RRCI) method [22], and estimated only for the *B*. *vaillantii* trawl fishery in the estuary. Further details on the stock assessment analyzes are provided in S1 Text.

#### Time series analysis

The trend analysis method was used to quantify the significance of trends of (i) the effectiveness of fishery management to reduce fishing mortality (F/F_MSY_ ratio) of the *B*. *vaillantii* trawl fishery in the estuary, considering the effect of all strategies, and (ii) the historical decline of catches of *B*. *rousseauxii* and *C*. *macropomum* sold in important urban centers as an overfishing indicator. The historical data of the F/F_MSY_ ratio of the *B*. *vaillantii* trawl fishery and the fish landings of *B*. *rousseauxii* and *C*. *macropomum* of each region were adjusted to the best model, considering the coefficient of determination (R^2^). The significance of trends was tested by the Mann-Kendall Test (MKT) for monotonic temporal trend, using the “Kendall” package. The variation in the capture between different periods along the time series was analyzed by the Kruskal-Wallis test (K-W) and a posteriori Dunn post-test for multiple comparisons. All tests were performed in R Statistical Software using the “Kendall” package for MKD test and the “Kruskal” and “Dunn” packages for K-W and Dunn tests and considering α = 0.05 [23].

## Results

### *Brachyplatystoma vaillantii* in Amazon River estuary

**Fig 5:**
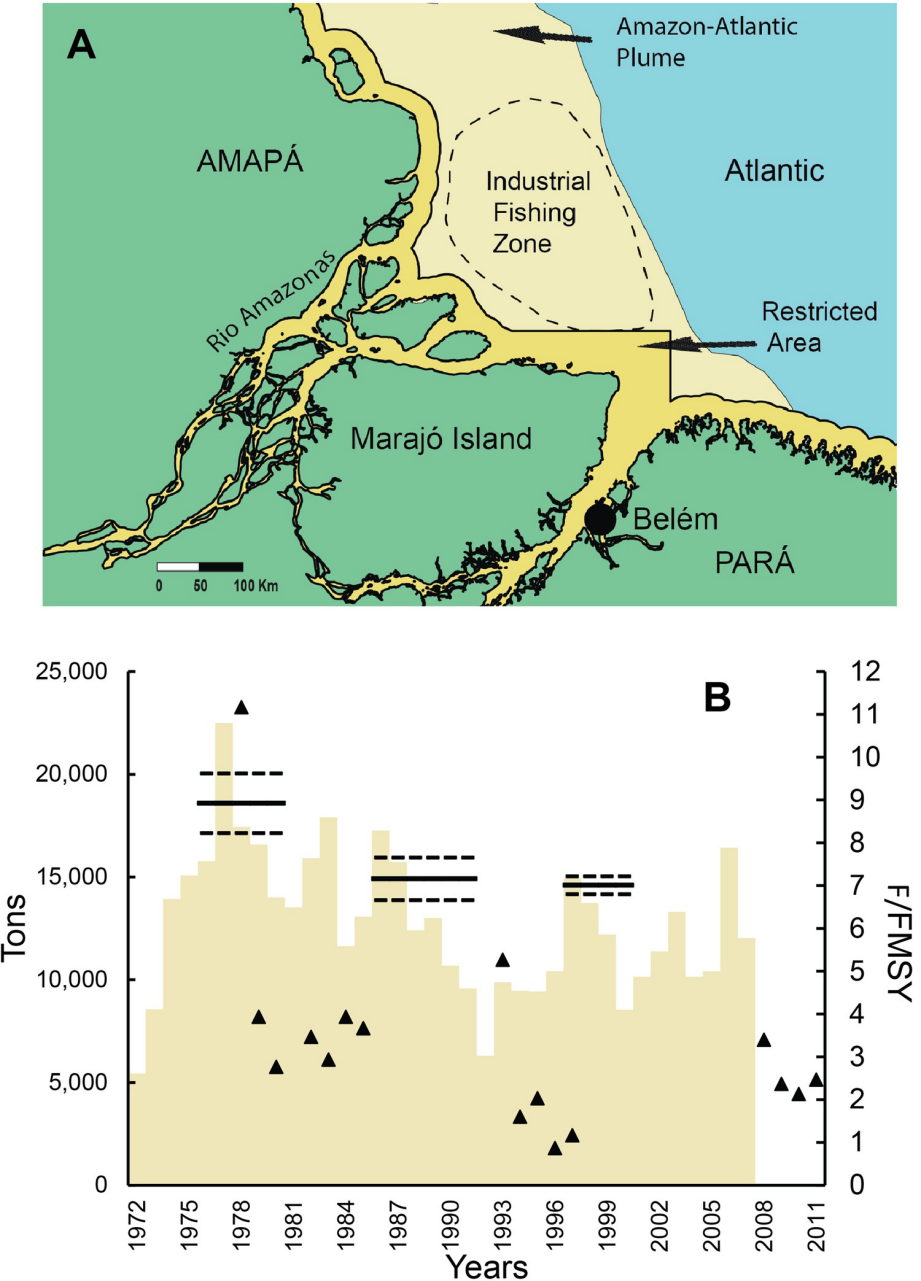
The goliath catfish fishery region and overfishing in the Amazon River estuary. (A) The industrial trawl fishing zone and the restricted fishing area based on legislation in force. (B) Annual catches (tons) of the *B*. *vaillantii* bottom pair-trawler fishing fleet in the Amazon estuary (yellow bars) available for the 1972–2006 period, combined with the Maximum Sustainable Yield proxies (tons) with 95% confidence limits (black line) and the F/F_MSY_ ratio (triangle).

The historical analysis of trawl fisheries for *B*. *vaillantii* represents a 34 year dataset between 1972 and 2006 (Fig 5 and S1 Table). We estimated the maximum yield in three periods when the catch reached a plateau after the fishing effort increased over time (S1 Fig), a condition that validates the assumption of the relative rate of the catch increase (RRCI) method (S1 Text) [22]. The 1976–1980 period corresponded to the first expansion of the trawl fishery of *B*. *vaillantii* in the estuary. The second period included an increase in the number of trawler fishing vessels from 43 to 66 boats in the period 1986–1991. The third period, 1997–2000, corresponded to the second expansion of the fishery after the historic lowest catch in 1992, when many boats began trawling together despite a total estuary industrial fishing limit of 48 boats [4, 24]. After these three periods, fishing effort was restricted by limiting the number of boats that could work together and by the establishment of a closed season.

The estimated MSY was very high for the first period (18,591 t/year) and similar for the last two years (14,602–14,915 t/year) (Fig 5 and Table 2). We consider the MSY estimate for the first period overestimated, because of the anomalous catch recorded in 1977 (22,486 t) after the fisheries were in operation for only five years. Our analyses indicate that *B*. *vaillantii* catches first exceeded MSY in the 1970s (Fig 5 and Table 2). *Brachyplatystoma vaillantii* has suffered high fishing mortality (F) for almost the entire industrial fisheries period since the 1970s and exploitation levels were at least twice F_MSY_ in 80% of the years (Fig 5). Considering all strategies to reduce fishing mortality, the effectiveness of goliath catfish management based on historical data showed a significant downward trend in fishing mortality just a few years after trawling began (Mann-MKT: tau = -0.393, 2-sided p value = 0.0382), suggesting that the measures applied had some effect on reducing fishing pressure.

**Table 2:** Maximum Sustainable Yield (MSY) estimates for *B*. *vaillantii*. RRCI’ smoothed relative rate of catch increase.

RCCI´ period	Year of MSY estimate	Approximate MSY (metric tons/year)	95% confident interval (metric tons)
1976–1980	1978	18,591	17,142–20,041
1986–1991	1987	14,915	13,879–15,951
1997–2000	1999	14,602	14,165–15,039

### *Brachyplatystoma rousseauxii* in the western Amazon region

Of the 5,482 *B*. *rousseauxii* specimens measured in the Western Amazon Region, 696 were from Madre de Dios River (Peru) in the period 2002–2004, and 4,786 from Ucayali-Urubamba River (Peru) in the period 2004–2005. Analyses and estimates of the population parameters of reproductive populations of *B*. *rousseauxii* in the Madeira (Brazil) and Ucayali-Urubamba basins, the two largest headwater regions, revealed different trends among these regions (Table 3 and S2–S5 Figs). The growth parameter of the fishes from the Madre de Dios River (department Madre de Dios) was slightly higher than that of the Ucayali-Urubamba River (L∞-_MD_ = 149.3 cm, and K_MD_ = 0.35 year^-1^ against L∞-_UU_ = 141.8 cm, and K_UU_ = 0.29 year^-1^). However, the current mortality rates influenced by fishing (Z and F) were quite different, with much higher values in Ucayali-Urubamba River (Z_MD_ = 1.18 year^-1^, and F_MD_ = 0.81 year^-1^ against Z_UU_ = 2.13 year^-1^, and F_UU_ = 1.8 year^-1^). The F/F_MSY_ relation indicates overfishing in the Ucayali-Urubamba River (F/F_MSY-UU_ = 2.73), and thus also in downstream fisheries exploiting stocks before they arrive near the Andes. An opposite situation occurred in the Madre de Dios River, where F/F_MSY_ indicates a “healthy” condition (F/F_MSY-MD_ = 0.91), at least before the Madeira dams blocked upstream migrations of *B*. *rousseauxii*.

**Table 3:** Population parameters of *B*. *rousseauxii* from Madre de Dios and Ucayali rivers.

Parameters	Madre de Dios	Ucayali-Urubamba
	(2003–2005)	(2004–2005)
L∞ (cm)	149.29	141.83
K (year^-1^)	0.35	0.29
M (year^-1^)	0.37	0.32
Z (year^-1^)	1.18	2.13
tc (year)	2.64	2.73
F (year^-1^)	0.81	1.80
F_MSY_ (year^-1^)	1.14	0.66
F/ F_MSY_ (year^-1^)	0.71	2.73

L∞: asymptotic length. K: growth coefficient. M: instantaneous rate of natural mortality. Z: instantaneous rate of total mortality. tc: age of entry into fisheries. F: instantaneous rate of fishing mortality. F_MSY_: fishing mortality at Maximum Sustainable Yield.

### *Brachyplatystoma rousseauxii* in the Brazilian Madeira River

**Fig 6:**
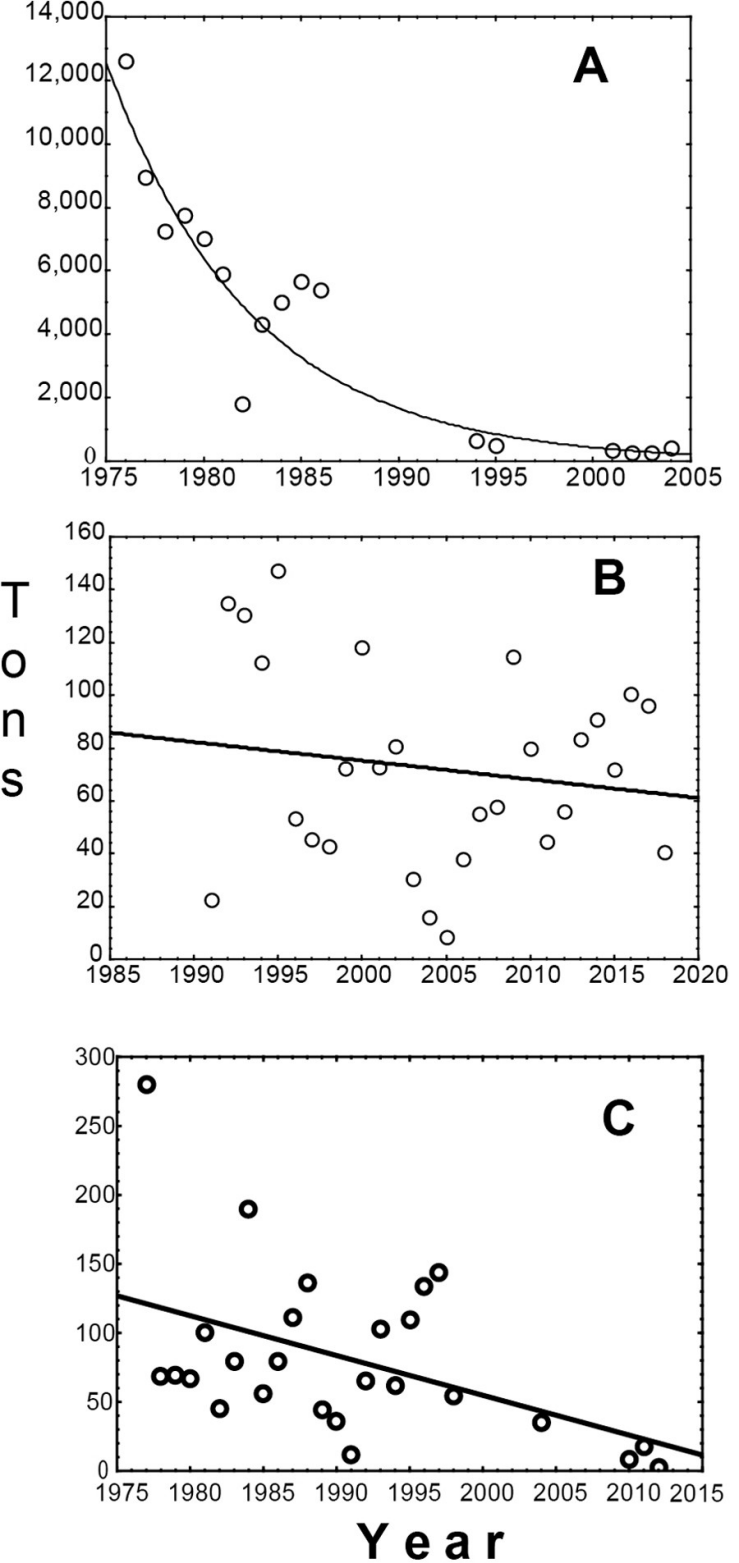
Historical trends in the capture of C. *macropomum* and B. *rousseauxii*. (A) Negative exponential trend adjusted to *C*. *mac*ropomum landings in Manaus (tau = -0.809; p<0.05); (B) Linear trend adjusted to *C*. *macropomum* landing in Tefé (tau = -0.032; p>0.05); (C) Linear trend adjusted to two periods of *B*. *rousseauxii* landings in Porto Velho (tau = -0.039; p<0.05).

Historical fishery data recorded at or near Porto Velho, Brazil, likely indicate temporal trends in the abundance of *B*. *rousseauxii* migrating upstream in the Madeira River. Initially, we tested the trend in the historical series of fish landings during the period 1977–2012. Next, we analyzed the variation of the annual fish production before and after the 1990 decade when stock depletion was first observed in other regions [2, 3, 25]. The first period considered only 1977–1989, the second period considered 1990–1998, and the third period 2004–2012 (Fig 6). There was a significant decrease in *B*. *rousseauxii* capture (MKT: tau = -0.039; p<0.05) during the entire period considered (Fig 6). The annual yield average of the three periods was 102, 80 and 16 tons, respectively, with significant differences between them (K-W H = 9,43, df = 2, p <0.05). Although catches in the first two periods were not significantly different, they were significantly higher than in the third period (Dunn post-test, p<0.05), which indicates a recent sharp depletion of the migratory stock in the Madeira Basin. Considering the large-scale migratory behavior of this species, a general decrease in the stock in the Madeira Basin could be due not only to overfishing but also be a consequence of the construction of the Santo Antônio dam that began in 2008 and was completed in 2011 [5, 26]. Overfishing and dams point to the deleterious synergy of these impacts on migratory fishes.

### *Colossoma macropomum* in the Central Amazon

**Fig 7:**
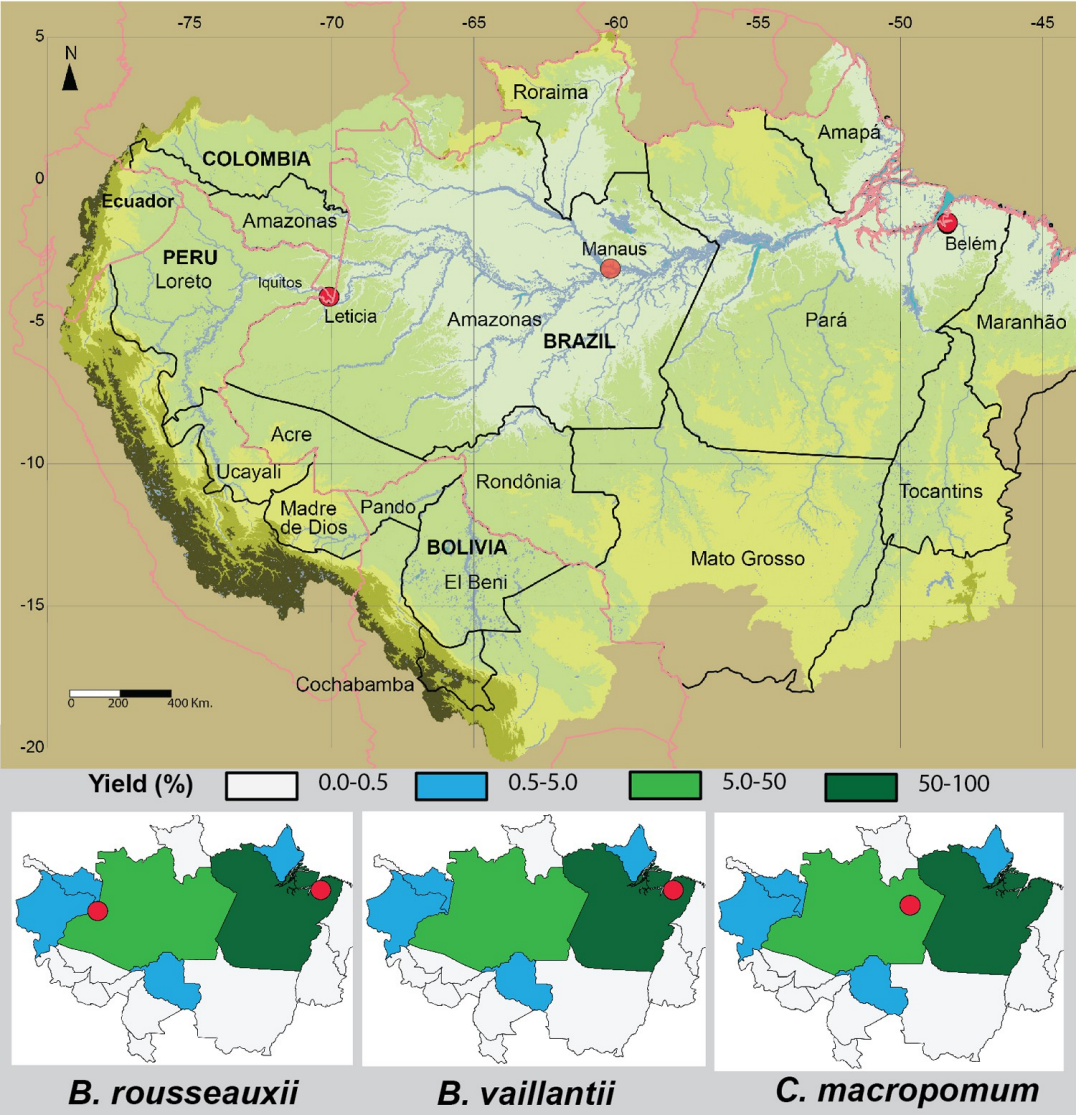
Main states or departments and cities associated with overfishing. Production in percentage categories. The highlighted cities are responsible for at least 50% of the production. Overfishing of the flagship species considered here needs to be controlled from the major urban ports that account for most of the landings.

The maximum annual historical harvest of *C*. *macropomum* is concentrated in the state of Amazonas, Brazil, which accounted for three-fourths of total landings in the Amazon (Fig 7). Manaus, the capital of Amazonas, Brazil, is historically the most important fishing port for *C*. *macropomum* and alone accounted for up to 83% of the total monitored catch of the species at the city’s principal market where most of the catch is commercialized (Fig 7 and S2 and S3 Figs). The maximum recorded catches in Manaus occurred in 1976, when ~13,600 tons were registered [18]. Records for subsequent years in this city show a clear downward trend in catches (MKT: tau = -0.809; p<0.05), dropping to 41% of the maximum 10 years later in 1986, and to only 3%, 29 years later in 2004, the last year Manaus landings were recorded [16, 17, 27–29] (Fig 6). In the intermediate city of Tefé (Brazil) between Iquitos and Manaus, and which is associated with the Mamirauá Sustainable Development Reserve, *C*. *macropomum* catches have oscillated between 147 and 8 tons, with no clear temporal trend (MKT: tau = -0.032; p>0.05). The Tefé catches, however, are not representative of the overall high pressure on the species since its maximum annual landings represent only 1% of Manaus maximum annual landings (Fig 6).

## Discussion

Commercial tropical inland fisheries are generally multi-species, multi-gear operations, especially in a large drainage basin area where fish diversity and environmental heterogeneity are elevated [30]. There are few examples where inland fisheries target single species or a few very similar species, such as the Nile perch (*Lates niloticus*) in Lake Victoria, various clupeids in Lake Tanganyika, and ilish (*Tenualosa ilisha*, Clupeidae) in the Ganges River [30–32]. At least the three fish species discussed in this article, and probably many more migratory species exploited in the Amazon, are also prime examples of highly targeted fisheries [4, 16, 31–33]. The commercial fisheries for Amazon goliath catfishes are widespread in the river basin, and they supply local fish markets and fish processing plants installed along the Amazon River and on the coast of the Amazon estuary. The goliath catfish fisheries occur mainly in the open waters of the turbid river channels and in fresh waters where the Amazon River enters the Atlantic [1, 34]. *Colossoma macropomum* fisheries occur in floodplain lakes, flooded forests, and river channels [16, 35].

There are insufficient monitoring data in the Amazon for adjusting statistically robust fish stock assessment models, a common challenge in inland fisheries that results in unrecognized and poorly reported declining freshwater stocks [36]. Although environmental degradation is of concern, intensive and/or extensive fishing pressure in the channels and floodplains of the Amazon River and those of its turbid tributaries appears to be the primary drivers for declining fish stocks [2–4, 7]. This includes recruitment overfishing, the most severe form of overfishing when reproductive populations are depleted, and growth overfishing when too many young fish are captured, either type of which leads to fishing mortality rates that exceed maximum sustainable yield [37]. A proactive approach to management should use the data available on stock assessment and the fish life history areas of migratory fishes to control overfishing in the Amazon. Larval density studies along with genetic analyses would provide a method independent of fisheries data to further strengthen stock assessments [38, 39]. Because the life histories of migratory species vary, each requires its own management strategy, and this is especially true for the species focused on here.

### Goliath catfish overfishing

There are seven species of goliath catfishes, and all are known to be migratory to some extent [8, 40]. The goliath catfishes are major river channel and estuarine predators found in the Amazon and Orinoco basins and some species in the coastal rivers of the Guianas [41]. Several of these species in the Amazon make the longest freshwater fish migrations in the world [8]. The migratory life cycle of *B*. *rousseauxii* can be at least 11,600 km between the Amazon River estuary and the far western Amazon, including the Andean Piedmont to at least 250 m in elevation [8, 10, 38, 40–42]. The migratory life cycle of *B*. *vaillantii* is less known but reaches to at least the Andes in the Marañón River of Peru [8, 43].

The general migratory routes of the targeted goliath catfishes considered here include the Amazon River and its turbid tributaries with headwaters in the Andes or nearby headwater areas in the Purus and Juruá basins. *Brachyplatystoma rousseauxii* also enters low sediment tributaries, such as the blackwater Negro River and clearwater tributaries arising on the Brazilian and Guiana shields. Statistically distinct size classes of *B*. *rousseauxii* recorded in commercial and research fishing clearly indicate an east to west increase in size, thus the separation of nursery and spawning regions [41]. Genetic analyses of goliath catfish stocks are not yet conclusive, though hypotheses suggest geographical variability for each species. To date, a single stock of *B*. *vaillantii* has been identified in the Amazon Basin [11]. Genetic studies of *B*. *rousseauxii* have shown mixed results, ranging from no genetic segregation between the populations in the estuary and near the border of Brazil, Colombia, and Peru [9], to at least three clusters in five locations sampled in the upper Madeira Basin in the Bolivian Amazon [44]. Although genetic studies are insufficient alone to rule out homing, otolith isotope analyzes indicate natal homing of *B*. *rousseauxii* in the upper Amazon [26]. A combination of tagging experiments and genetic and otolith analyses will be needed to determine conclusively if homing occurs.

Industrial and artisanal fishing operations exploit goliath catfishes in the Amazon River estuary, whereas only artisanal fishing takes place in inland waters. The industrial fishing fleet in the estuary is composed of sea-worthy vessels that use bottom pair-trawlers that operate in fresh waters up to 50 km offshore of the Amazon River mouth [1, 45–48] (Fig 5). Artisanal fisheries for goliath catfishes across the Amazon sell fish to urban centers and refrigeration plants and employ a combination of drifting gillnets, seines, longlines in river channels, and gaffs at cataracts [49, 50].

Fishery subsidies and/or export markets were external drivers that historically contributed to goliath catfish overfishing. As early as the 1990s, *B*. *vaillantii* was reported overfished in the estuary [4], as also was *B*. *filamentosum* in the western Amazon [7]. *Brachyplatystoma filamentosum* spawns across the Amazon in great contrast to the long-distance migratory species, *B*. *rousseauxii* and *B*. *vaillantii*, whose nurseries and spawning areas are widely separated at the farthest eastern and western regions of the basin. Subsidies and export markets are common characteristics of goliath catfish fisheries in Brazil and Colombia, the two most important countries exploiting these species in the Amazon. In the early 1970s, industrial-scale fishing companies operating in the estuary, and subsidized by the Brazilian government, focused on *B*. *vaillantii* because of its export mainly to the United States [1, 45, 47]. In Colombia, between 1978 and 1982, increased coca production for the international cocaine market led to an economic boom that indirectly stimulated the catfish fisheries of the Amazon, Putumayo, and Caquetá rivers to supply fish to national markets [34, 50]. The Colombian port city of Leticia (department of Amazonas) served as the main base for these export fisheries based on artisanal operations. Subsidies and export markets also led to the establishment of fish processing companies in the Brazilian states of Pará and Amapá and the Colombian department of Amazonas. Other than the trawl fishery in the estuary, investments were made by national and regional (i.e., department or state) governments, without concomitant catch monitoring.

The Brazilian bottom pair-trawl operations in the Amazon Estuary represent a rare example of a single-species and industrial-scale freshwater inland fishery in the tropics, given that the main target is *B*. *vaillantii*, whose catch represents 76% of recent trawler production, followed by the 7% catch of *B*. *rousseauxii* [51]. Trawler bycatch has been reported but there are few details on how much is commercialized or discarded [52]. Initial government-based studies indicated MSY above 20,000 t/ year [24]. However, our data and recent estimates of MSY of *B*. *vallantii* indicate that the initial MSY was highly overestimated [53]. By 1992, the year with the lowest catch (6,299 tons), overfishing resulted in the collapse of *B*. *vaillantii* as an export species. It fell from the third most important marine or freshwater fish species exported from all of Brazil in 1986 and 1987 to no longer appearing in export lists [4, 24, 41]. The strategies to reduce fishing mortality have been focused on the minimum mesh size of the cod-end of the trawl net, the size of the fishing fleet, protected areas, and closed seasons [46]. Our data indicate that these strategies reduced mortality, although not enough to prevent overfishing.

Overfishing of *B*. *rousseauxii*, the fish species undertaking the longest migrations in the Amazon Basin, was reported for the Caquetá River, Colombia, in the period 1995–1998 [2] and Amazon River, Brazil, in the period 2002–2003 [3, 25]. To reach the western Amazon, the migratory catfish populations must first avoid capture along a several thousand-kilometer-long route where commercial fisheries operate in the Amazon River and its tributaries in Brazil, Colombia, and Peru [1, 25, 34, 47]. In contrast to *B*. *vaillantii*, after migrating upstream from the estuary, *B*. *rousseauxii* does not return to it, though the juveniles of both species born in the western Amazon migrate back to the estuary. *Brachyplatystoma rousseauxii* is heavily exploited during its long 2–3-year upstream migration to the western Amazon, thus making it vulnerable to both growth overfishing of young fish by the trawler fleet in its estuary nursery and recruitment overfishing of mature stocks upstream [2, 3, 25].

### *Colossoma macropomum* overfishing

*Colossoma macropomum* is the second largest scaled fish in the Amazon and one of its most highly prized food species and cultural-culinary biodiversity symbols. Historically, *C*. *macropomum* reached 100 cm TL and lived up to 65 years of age [16, 19]. The species is exploited by fishers in nine states or departments, ranging from the eastern Amazon near the estuary in the states of Amapá and Pará in Brazil to the far west in the departments of Cochabamba and Beni in Bolivia and Madre de Dios and Ucayali in Peru. In the 1970s, it was the most important species commercialized in Manaus, the Amazon’s largest inland fish market [17, 18]. It is the iconic symbol of the flooded forests because of its fruit- and seed-eating habits and its role in dispersing many seed species that pass through its intestinal system and remain viable for germination [16, 54–56]. The species is widely distributed in the Amazon Basin and found in all river types. Its nurseries, however, are found mostly in floodplain lakes of turbid rivers with headwaters in the Andes, and include the Amazon, Madeira, Purus, and Ucayali [57]. When water levels begin to fall rapidly, adult fish largely move from the floodplains to the river channels where migrations take place at various intervals until the fish spawn with rapidly rising river levels. After spawning, the fish migrate to flooded forests of all river water types. In addition to fishers’ and our field observations, *C*. *macropomum* migrations have been verified by ichthyoplankton fishing in river channels where the newborn can be carried 400–1,300 km downstream before entering a floodplain nursery [16, 58, 59]. Thus, it can be assumed that adults, on average, migrate a similar distance upstream during the six-month period or longer that they reside in Andes-Amazon River channels before spawning.

The widespread use of gillnets beginning in the 1960s made it possible to catch large quantities of *C*. *macropomum* in floodplain lakes and flooded forests [35]. The increased use of purse seines also greatly increased fishing pressure on adults found in river channels during the low water periods when these fish are migrating or seeking refuge along woody shore areas. Immature, 2-3-year-old *C*. *macropomum* are among the largest fishes exploited in the open waters of floodplains [1]. Although principally a fruit- and seed eater after about 2 years of age, young *C*. *macropomum* are highly zooplanktivorous and feed in open waters of floodplain lakes where they are vulnerable to both gillnets and seines [16, 18, 60]. As adult *C*. *macropomum* became rarer in the 1980s, local communities increased their catches of young fish, an occurrence that was and still is easily observable in urban markets in Brazil and Peru (Fig 4). Once *C*. *macropomum* adults became relatively rare, an adult fish of 12 kg or larger sold for $75–100, thus compensating for the low catch per unit of effort required to capture it. Additionally, as passenger-freight boats began to be used to transport ice to and fish from far-flung communities along the rivers, catches could be delivered from hundreds of km away on a weekly basis [61]. This new type of fish transportation not only replaced urban-based large fishing boats that previously accounted for most of the *C*. *macropomum* catch, but also increased the maximum distance from urban markets where captures were made. In the case of Manaus, the distance expanded from perhaps 500 km in the 1970s [18] to at least 1,000 km after the year 2000, a phenomenon that has been referred to as the ‘rainforest metropolis defaunation shadow’ [62].

The most robust datasets for *C*. *macropomum* stock assessment are from commercial fisheries, though with serious hiatuses. Empirical abundance data independent of fisheries data is available for a few locations and measured by ichthyoplankton densities [63]. The life history of the species is reasonably understood, including life history areas [14, 16], fish size range [17, 64], age [16, 17, 65, 66], general migration patterns [57], and reproduction [16, 19, 58, 67, 68]. Our analyses rely mostly on historical fisheries data to detect overfishing trends, especially as indicated by data for the large Manaus market that accounts for most of *C*. *macropomum* catches.

Due to its flooded large forests and floodplain lakes on which *C*. *macropomum* depends [14], the central Amazon is the center of *C*. *macropomum* fisheries. The first yield-per-recruit (Y/R) study of *C*. *macropomum* covered the period 1976–1978, and that included a large area of the Central Amazon, concluded that there was no growth overfishing for this period [17, 69]. Later studies, however, indicated a 50% reduction in catch per unit of effort between 1979 and 1986 and that the *C*. *macropomum* stock had been overfished in the Central Amazon [29]. This coincided with a period when the Manaus fishing fleet expanded its fishing river distance from 1,700 km to 3,700 km [70]. Subsequent Y/R studies in the 1980s and 1990s also indicated *C*. *macropomum* overfishing in the lower Amazon centered on the city of Santarém [6, 71, 72], in the Manacapuru tributary just west of Manaus in the Central Amazon [73], and farther west centered on the city of Tefé near the Amazon (Solimões) River [74]. A large study of length frequency distribution of *C*. *macropomum* in the Manaus market between1993 and 2006 further indicated growth overfishing of the species [64].

Female wild *C*. *macropomum* mature as small as 45 cm, though most are larger than 55 cm [16]. The 55 cm maturity baseline, which includes fish about four years of age [65, 66], has also been used as the legal limit for commercial catches since the 1970s. By the 1990s, over 90% of *C*. *macropomum* sold in urban centers along the Amazon River were fish less than 55 cm, which was the minimum catch length legally allowed in the 1977–1978 period. By the 2007–2008 period nearly all *C*. *macropomum* sold in urban markets were illegal (Table 4). Recent larval studies in the Ucayali and Marañón rivers in Peru [39] and in the Solimões River in Brazil [75] revealed alarmingly low densities of *C*. *macropomum*. The absence of *C*. *macropomum* breeding adults is now so pronounced that fishers report they no longer detect reproductive adults of the species in the lower Amazon [76].

**Table 4:** *C*. *macropomum* size composition in three fishing ports of the Amazon.

Fishing port	Year	ML	Lc	PL	Source
Manaus	1977–1978	69	55	0%	Petrere 1983 [17]
Tefé	1993	43	39	93%	Costa et al. 1999 [74]
Santarém	1992–1993	40	28	>90%	Isaac & Ruffino [77]
Manacapuru	2007–2008	31	17	98%	Campos et al. 2015 [73]

ML (cm): mean lengths in the catch, Lc (cm): mean length of first capture; PL: capture percentage below legal limit size.

*Colossoma macropomum* contains a single and large panmitic population along the Amazon River and genetically differentiated populations in tributary headwaters, especially in the Madeira Basin above the cataract stretch in Rondônia, Brazil [78]. The low inter-population genetic differentiation in the Amazon River and lower and middle courses of its tributaries is probably related to the large-scale migratory behavior of the species that acts as a homogenizing agent [79]. Most of the commercial catch consists of this genetic stock. The widespread evidence of *C*. *macropomum* overfishing indicates that the large homogenous population is under a severe threat of collapse. Above the Madeira Rapids in Bolivia, earlier data indicated a relatively unexploited but minor stock compared to that in the Central Amazon [19, 80], but the status of the species in Bolivia is unclear now.

As a result of overfishing and the popularity of *C*. *macropomum* in aquaculture, it is either the most important or second most farmed species in the Amazon [16, 81]. Though data are minimal on farmed fish species in the Amazon, the total *C*. *macropomum* production sold in Manaus is probably greater than maximum wild catches recorded in the mid-1970s. It should not be assumed, however, that aquaculture has reduced fishing pressure on wild *C*. *macropomum*. Farmed *C*. *macropomum* are sold at about 1.5–3.0 kg, that is, immature fish and, as with wild fish of the species, are destined to the middle and upper economic classes. Large adults are only available from wild fisheries and have commanded the highest prices of any fish species since the 1990s, thus recompensing the effort invested in catching them despite their rarity [16]. Finally, despite the contribution from aquaculture, young wild *C*. *macropomum* also continue to be exploited across the Amazon, including in protected areas.

### Impacts other than overfishing

The potential major impacts on fisheries other than overfishing are wetland and upland deforestation, dams, and water quality degradation. Though upland deforestation and water quality degradation could eventually surpass overfishing, wetland deforestation, and dams as the principal impacts on fisheries, at present these impacts are less limiting ecologically. Climate change is also expected to cause major hydrological changes [e.g., 42, 82]. In addition to overfishing discussed above, here we address dams and wetland deforestation as the two impacts that require immediate mitigation for robust fisheries management.

#### Dam impacts

There are no dams on the Amazon River, and none are proposed, but its largest tributary, the Madeira, has been impounded in two places and at least one more large upstream dam is planned. In great contrast to *B*. *vaillantii and C*. *macropomum*, *B*. *rousseauxii* has been highly impacted by the Madeira hydroelectric dams in the state of Rondônia, Brazil. Unlike other goliath catfishes, upstream migrations of *B*. *vaillantii* in the Madeira River largely stopped at the second cataract, the Cachoeira de Teotônio, just upriver of Porto Velho [49]. No fisheries for *B*. *vaillantii* have been reported for Bolivia or Peru in the Madeira Basin before or after the dam construction [83, 84]. In contrast, ripe *B*. *rousseauxii* ready to spawn and their eggs and larvae have been verified in the Madre de Dios, Beni, and Mamoré rivers of the upper Madeira Basin, thus including most of its vast headwaters as reproductive locations [5, 8, 38, 85, 86]. The Madeira is the Amazon Basin’s largest tributary in both discharge and headwater area [87], thus was one of the most extensive spawning areas not only for *B rousseauxii* but for several other goliath catfishes as well (*B*. *platynemum* and *B*. *juruense* and *B*. *tigrinum*). The two Madeira dams are about 1,200 km downstream of Andean headwaters where *B*. *rousseauxii* spawns, thus nearly one -third of its Amazon Basin spawning headwaters are now inaccessible since 2012 [87, 88]. The Bolivian and Peruvian populations of *B*. *rousseauxii* in the Madeira Basin are rapidly disappearing for lack of recruitment, and thus also fisheries for the species [5].

The other large dams on Brazilian Shield rivers, the Tocantins and Xingu, appear to have had no serious impact on the goliath catfishes and *C*. *macropomum*, though they may have impacted other species that migrate within these basins [e.g., 89–92]. The Tapajós is the last of the large Brazilian Shield rivers whose mainstem is undammed, and local information indicates there might be a small *C*. *macropomum* population in the middle course of the tributary that perhaps reproduces there and that supports small-scale fisheries [93]. Based on fisheries, the Madeira dams have had little impact on *C*. *macropomum* as the species is poorly adapted to pass torrential cataracts [49] and impacts on the seasonal hydrological regime on which it depends downstream of the dams have been modest [94]. The genetically distinct population in Bolivia above the rapids further indicates a lack of large-scale upstream migration even before the dams were constructed [78].

#### Wetland deforestation

Large-scale deforestation of wetland forests is most pronounced along the Amazon River from just east of the Negro River to western Marajó Island in the estuary. The floodplains in much of this area suffered deforestation beginning with the jute boom in the 1930s and later with widespread zebu cattle and water buffalo ranching since at least the 1960s [95, 96]. There are no empirical indications of how floodplain deforestation might have impacted goliath catfish migrations, though it is reasonable to hypothesize that their prey species that migrate seasonally from the floodplains to the river channel have been affected [41]. Floodplain deforestation has probably impacted *C*. *macropomum* in the eastern Amazon River where there are large floodplain lake nurseries and once extensive floodable forests [97–99]. Because ecological fisheries studies were not made before the 1970s during the major period of floodplain deforestation, it is difficult to parse the relative impacts of overfishing and floodplain deforestation. Theoretical deforestation models that included impacts on *C*. *macropomum* in the lower Tapajós River indicated a precipitous decline in the biomass of the species [100].

## Proactive management recommendations

### Reinstate government responsibility to collect urban fishery market data

The importance of collecting fisheries data in the Amazon dates to early Portuguese colonies in the 17^th^ century and was emphasized during the rubber boom at the end of the nineteenth century in the first major overview of the region’s fisheries [101]. Nevertheless, in general, data collecting has not been continuous, even after the exponential growth of the fisheries beginning in the 1960s. Early modern efforts to collect fisheries data in the 1970s and early 1980s were largely led by individuals with the support of various governmental or non-governmental institutions, and give a snapshot of the geographical scale, species exploited, catches, and fishing effort before overfishing became a widespread problem in the Amazon [18, 49, 102, 103]. These studies emphasized and demonstrated the importance of urban market and refrigeration company data collected in major cities for managing fisheries based on migratory species. In the last two decades some continuous data has only been collected by Peruvian and Colombian fisheries agencies and locally by the Mamirauá Institute located in Tefé, Brazil (S1 Text).

Unfortunately, the lack of continuous monitoring of fisheries across the Amazon over the years compromised the assessment of stocks and the establishment of adequate management measures to detect and prevent overfishing. Despite the Amazon’s large basin size and ever-increasing infrastructure development, academics, NGOs, and financial institutions still favor small-scale fisheries initiatives, usually under the social umbrella of community-based management, to guarantee at least the livelihoods of the human populations that live along floodplain lakes and river channels [99, 104–108]. It is unclear to what extent these initiatives have been successful at regional scales for fisheries management of large and widespread stocks. Their actions have had well documented success in the recovery of relatively sedentary species, such as *Arapaima gigas* [104], and socially for the organization and transfer of fishing rights from outside users to rural communities. Although community-based management can protect sedentary stocks and favor production for floodplain lake fishers, this strategy alone is not adequate for managing long-distance migratory species at the large scales of migratory fish life histories. To manage the migratory species at sustainable fishing levels, and thus the most important commercial species, it is necessary to maintain continuous data collecting in urban markets for at least the most important fisheries. These data can then be used for routine stock assessments, to detect overfishing, and support the agencies responsible for managing the fishery resources to make the most appropriate decisions to ensure the sustainability of the fisheries.

Successful fisheries management worldwide is associated with solid monitoring and robust stock assessment, and especially for larger stocks that receive far more management attention than smaller and unassessed stocks [109]. The most basic data for fishery management are historical and contemporary catch and effort, size composition, and sexual maturity of the catch [110]. Despite the large-scale increase in the exploitation of fish stocks, Brazil, the largest country with the largest area in the Amazon Basin, has not collected fishery data in most urban centers, including the largest, Manaus, since 2004. Furthermore, the use of statistics for setting science-based management regulations for Amazon fisheries continues to be ambiguous due to lack of confidence in governmental regulatory implementation and, as mentioned above, the relinquishment of governmental fishery responsibilities to local community-based management alone. Proper assessment of stocks could be reinforced if researchers, fishers, NGOs, consumers, and other stakeholders insist on the need for reinstating governmental responsibility to collect data and assess urban markets, ultimately providing the international framework required to understand overfishing at migratory fish life history scales.

### Reduce fishing effort through enforced market regulations

Given the large areas involved in the Amazon and the precarious environmental infrastructure to patrol the interior, the most effective option to manage fishing effort is through enforced market regulations, such as size limits and closed seasons of fish sold in urban markets or to refrigeration companies. This obviously assumes that if the fishers cannot sell prohibited species, they will not capture them in large quantities. An argument against targeting species might be that in multi-species and multi-gear fisheries it is extremely difficult to target single species. The three species discussed here, and most of the other important migratory species, however, are highly targeted because of some combination of their large size, market and cultural values, and local fishers’ intimate knowledge of their life history locations and seasonal migratory behavior.

Although the results of *B*. *vaillantii* and *B*. *rousseauxii* overfishing are worrying, the fishery collapse for these species can be prevented and stocks rebuilt within a decade if fishing mortality is rapidly reduced below the fishing pressure that gives the MSY [111]. The most effective way to reduce fishing pressure on goliath catfishes is to reduce the fishing effort of the industrial fishing fleet in the estuary [24, 53, 102] and to adopt a capture quota or total allowable catches (TACs) for the Amazon Basin. The TACs are one of the most positive measures for rebuilding overfished stocks [21], and can be established at the main landing sites, such as at fish processing plants and markets in the estuary and along the inland rivers in the Amazon [47]. Local managers have had some success in reducing fishing pressure in the estuary, but not enough to control catches by the trawler fleet. Unfortunately, the necessary recovery time before expected collapse is running out. Finally, the lack of monitoring for more than a decade by Brazilian fishery agencies is ominous for the management of estuarine trawl and other fisheries. The most daunting challenge facing the management of *B*. *rousseauxii* is the exploitation of mature migratory fish in Colombia and Peru in the western Amazon that has led to recruitment overfishing. The loss of major spawning areas in the Madeira Basin because of the construction of dams blocking upstream migrations has added further to declining *B*. *rousseauxii* stocks in the Amazon Basin. A limited fishing period during the main spawning period of the species near the Andes would benefit the fisheries of Andean countries and Brazil [8, 25].

*Colossoma macropomum* has been so heavily overfished that a fish market moratorium will be required to rebuild its stock [112]. This will also require collecting separate data for wild-caught fish and those from farming operations to detect to what extent young wild fish are being exploited. Rather the moratorium is spatially and/or temporally implemented would not necessarily require a complete closure of the fishery [113]. How long the moratorium should be in place would depend on abundance data other than fisheries since prohibiting its sale also implies that its regeneration could not be detected in urban markets where the species was prohibited. The easiest, cheapest, and most quantitative method to do this would be through ichthyoplankton surveys in river channels during the spawning season [67, 68].

### Remove subsidies

Fisheries strongly driven by export markets are usually difficult to control because economic pressure contributes to removing the last remnants of traditional local management [114]. Export markets from the Amazon are now restricted to each country and most important in Brazil and Colombia. Fishing subsidies directly or indirectly contribute to fleet overcapacity and stock overfishing [115]. Subsidies and export markets in Brazil and Colombia were the main incentives to the exploitation of goliath catfishes in the Amazon, which is reflected by the maximum annual fishery production of *B*. *rousseauxii* and *B*. *vaillantii* in each state or department (S2 Table). The Brazilian states of Pará and Amazonas and the Colombian department of Amazonas represent 84% and 95%, respectively, of total production of *B*. *rousseauxii* and *B*. *vaillantii* in the Amazon Basin. The cities of Belém in Brazil and Leticia in Colombia are responsible for at least 50% of all yields of these two species (Fig 7).

Currently, Brazil still maintains fishery subsidies in the form of unemployment benefits to artisanal fishers during the closed fishing season [116], and a tax exemption on the purchase of diesel oil for fishing vessels [117]. Fishing subsidies have had negative effects in the Amazon in both the industrial and artisanal fisheries. The subsides weakened market forces that might have curbed fishing pressure. Most ominous were federal fishery subsidies associated with the Closed Fishing Season Law in effect since 2003 in Brazil, a supposedly legal means to decrease fishing pressure that had the opposite effect. The law encouraged fishers to participate in the fisheries solely for government subsidies without any meaningful monitoring of catches such as for the highly threatened *C*. *macropomum* [118].

### Unite urban and rural fishery management

The synergies between overfishing, hydroelectric dams, and floodplain deforestation present immediate major challenges for fisheries management in the Amazon, and especially for migratory species [10]. Hydrological changes due to climate change present further challenges to overall impacts in the long term [64, 119]. The migratory species are especially vulnerable to overfishing in their floodplain or estuarine nurseries and in river channels when they are schooling and migrating. The three migratory species considered here represent only a trio among dozens of commercial fish species that migrate in river channels, but their decades-long overexploitation is leading to the potential tipping points of stock collapse. We believe it would be a mistake to accept the collapse of these species as an unavoidable consequence of the fishing-down concept where larger species, often predators, are overexploited and the commercial fisheries turn to smaller species at lower trophic levels. If these species are not preserved, we believe that incentives to protect other targeted migratory species and the wetlands on which they depend, will be further weakened, and exacerbate the need for fisheries management at the ecosystem scale. Such iconic species have high cultural and other values that need to be factored into management strategies [120].

Fisheries managers have various options to control overfishing, though no one option is ideal for all fishing operations and species in the Amazon [69, 121]. Due to limited governmental intervention in the fisheries, NGOs and academics launched community-based management strategies in the 1990s. The major oversight of community-based management was the failure to recognize the overwhelming importance of migratory species (80+% of total catches) in commercial fisheries, and thus the necessity to concomitantly enhance fisheries data collecting in urban centers that are necessary to detect regional fishery patterns, such as overfishing and stock collapses. Furthermore, the lack of governmental fisheries management at urban levels resulted in a pessimistic scenario of the ability to manage fisheries through enforced market regulations based on annual statistics of catches and fishing effort.

Fisheries management in the Amazon should not be binary as either directed solely by governmental authorities or solely by hundreds of local communities largely operating independently along the rivers. There are few examples where local communities have attempted to manage *C*. *macropomum* overfishing. Controlled fishing zones seemed to have some positive effect on local *C*. *macropomum* populations where no-take floodplain lakes provided recruits of larger fish in the area of the Piagaçu-Purus Sustainable Development Reserve in the lower Purus River of Brazil [122]. It is unlikely that such an approach would be successful across the large commercial fishing area as it would be highly challenging to implement and monitor. A more dynamic approach is required where the two complementary components of urban regulated fisheries and community-based management are developed and interact together through regional fishery associations or the like. Some progress to this end has been made at small regional scales, such as at the Mamirauá Sustainable Development Reserve in the central Amazon that accompanies fishery catches in the small nearby city of Tefé to inform the Reserve’s management. A similar situation occurs in the Pacaya-Samiria National Reserve near the confluence of Marañón and Ucayali Rivers in Peru, where the governmental agencies of production (DIREPRO) and conservation (Servicio Nacional de Áreas Naturales Protegidas- SENAMP) share the registers of fish production in the protected area. This coupling of urban and rural initiatives has less continuity outside of extractive reserves and indigenous territories. Although encompassing large areas, even together, the ongoing local efforts are insufficient to manage long-distance migratory species within their large life history areas to supply the high demand for fish in urban centers.

### Foster interstate and international agreements

The definition of migratory species is somewhat subjective, as nearly all species migrate to some extent, and depending on the species, ranging from a few kilometers to thousands of kilometers during their life histories in the Amazon [123]. One category is long-distance migratory species that for the Amazon have been defined as total life cycle movements greater than 1,000 km in river channels [8, 14]. The Amazon Basin includes five countries relevant to long-distance migratory fish conservation and seven states or departments in four countries that exploit these species (Fig 1). The transnational migratory behavior of goliath catfishes with nurseries and reproductive areas in different states or departments and countries presents major political challenges to the management and conservation of these species. The most important secondary administrative units where overfishing takes place are the Brazilian states of Pará, Amazonas, Rondônia, and Amapá, the Colombian department of Amazonas, and the Peruvian departments of Loreto and Ucayali (S3 Table). These jurisdictions are largely responsible for allowing excessive fishing pressure that has resulted in overfished stocks of the long-distance migratory *B*. *vaillantii*, *B*. *rousseauxii*, and *C*. *macropomum* and the local migratory *B*. *filamentosum*, the last which was reported seriously overfished in the early 1990s [7].

To date, fishery policies across jurisdictions are not coordinated, though the importance of migratory fish species is now recognized, and studies are increasing [10]. Brazil occupies the largest area of the Amazon Basin, but hydrographically it is downstream of all other countries. At the country level, the western headwater region associated with the Andes embraces four countries: Bolivia, Colombia, Ecuador, and Peru. With 15 secondary administrative levels (states, departments, or provinces), the Andean piedmont region of the Amazon Basin is far more fragmented geopolitically than the lowlands. Although all Amazonian countries except Colombia are signatories to the Convention on the Conservation of Migratory Species of Wild Animals, no Amazonian freshwater migratory fish species are listed in the Convention. Other possible international organizations that could help stimulate fishery management across countries include the Food & Agriculture Organization (FAO), the Organization Cooperation Treaty Organization (OTCA), the Ramsar Convention, and the Convention on International Trade in Endangered Species of Wild Fauna and Flora (CITES) [15].

### Mitigate the Madeira dams

The Madeira dams represent the first major infrastructure mitigation challenge to fisheries management linked from the Andes to the Amazon estuary. A fish bypass was constructed at the first downstream dam, Santo Antonio, though it does not function as planned [124]. Because legal contracts require migratory fish passage through the Madeira dams, the bypass options need to be investigated further. We believe it would be a mistake to forgo further mitigation of goliath catfish passage through or around the Madeira dams as legal agreements require it until successful, and to not do so would set an ominous precedent for the future.

### Monitor floodplain deforestation

Floodplain deforestation directly impacts species that use the floodplains as nurseries, as spawning habitats for many species, and for growth of older fish, such as feeding seasonally in flooded forests [97, 122]. The historical trend of large-scale floodplain deforestation in the Amazon is east to west along the Amazon River, and if it continues at a similar rate in the next 3–5 decades then it will reach as far west as the Andean countries.

Floodable forests are the most common natural wetland type of floodplains of large Amazon rivers and the estuary [125]. Floodplain deforestation analyses have often been overlooked or subsumed into upland deforestation, yet the impacts on wetlands are far more drastic *per area* because they affect directly aquatic, arboreal, and terrestrial biodiversity. The amount of flooded forest correlates with fishery production and fish biodiversity because the floodplains are nurseries and feeding areas for most species that migrate in the river channels [14, 98, 99]. More than 1,000 km of the lower Amazon River floodplain has been largely deforested and is now in relatively species-poor secondary forest or open herbaceous wetlands where forest existed before. Although beyond the purview of this paper to suggest specific interventions to decrease wetland deforestation, direct governmental intervention is necessary. Especially helpful would be a clear separation of upland and wetland deforestation to call more attention to the importance of both types of forest in the aquatic ecosystems of the Amazon. The impacts of upland deforestation, especially in headwater areas near or in the Andes, are also highly relevant to the life history of long-distance migratory fishes because they protect watersheds, but this relationship is poorly understood [38]. Upland deforestation is further complicated geopolitically because Andean western headwaters are shared by four countries (Bolivia, Peru, Ecuador, and Colombia), but with the largest country, Brazil, downstream of all of them.

### Include overfishing as part of ecosystem level impacts

Though little investigated, overfishing of freshwater migratory fish species can have ecosystem-level consequences on species other than just the exploited species [126]. For example, *C*. *macropomum* is an important seed disperser in flooded forests of the Amazon, and not only because of the large number of plant species whose fruits it eats [127], but also the long distances that it disperses seeds [56, 128]. Thus, overexploitation probably disrupts an ancient coevolutionary relationship between *C*. *macropomum* and Amazonian plants, and especially because of the large size of the fish that is associated with a higher level of seed dispersal [127, 129, 130] The decrease in *C*. *macropomum* biomass through overfishing also eliminates one of the most important seed dispersal agents of the Amazon’s flooded forests. Many other migratory species exploited in fisheries are also seed dispersers [127].

## Conclusion

In this paper we present the status of three important migratory fishes in the Amazon, and we argue that governmental intervention is necessary if the Amazonian commercial fisheries based on migratory fisheries in general are to be managed in any sustainable manner. The overwhelming importance of medium- to large-sized migratory fishes in commercial catches, and their popularity regionally, means that local communities alone should not be assigned the full responsibility to manage them. The only practical way to assess and manage migratory species in the long run in an area as large as the main commercial fishing area in the Amazon is at market sites where enforced regulations can control fishing effort by prohibition of the sale of wild overfished species. All Amazonian countries have collected fishery data at least in some years since the 1970s and passed regulations to control fishing effort, thus there already exists, at least tacitly, a policy and regulatory framework for governmental intervention in the fisheries.

In the case of estuarine fisheries based on *B*. *vaillantii*, a select group of medium and large Brazilian companies operating in the estuary are required to follow all Brazilian environmental legislation and regulations. Governmental intervention, in this case, would be direct on these companies so that the profitability of the companies is maintained through sustainable catches. Overfishing of adult *B*. *rousseuxii* could be controlled mostly in the western Amazon, as overfishing of the young found only downstream is of less concern. Based on historical fisheries data of the large urban markets, *C*. *macropomum* has already reached the point of collapse from overfishing in its most productive region, and it is critical that the Brazilian states of Amazonas and Pará suspend the capture, transport, commercialization, and storage of wild fish of this species for a period of at least four to five years. During this period, monitoring recovery of stocks can be undertaken with ichthyoplankton sampling in river channels.

The management of the three species considered here has implications beyond just their sustainability. Their management would represent a paradigm shift where the governments assume their legal responsibilities in fishery management. These responsibilities include regulation enforcement, data collecting, inter-jurisdictional cooperation to protect migratory species at realistic life history scales, mitigation of the Madeira dams to assure goliath catfish passage to the largest western headwater region, and recognition of monitoring and managing wetland deforestation for the protection of fish and other aquatic and terrestrial biodiversity.

## Supporting information

S1 Fig Analysis of the catch increase smoothed RRCI (RRCI’) for the *B*. *vaillantii* bottom pair-trawl fishery. (A) Changes over time in the RRCI of *B*. *vaillantii* captured in the Amazon River estuary during a 34-year period (1972 to 2006). Years corresponding to significant changes in the fishery were not used to estimate the maximum yield and are represented by a black dash. Data with minor changes used for the regression are represented by diamonds, squares, and circles for each catch trend of years. (B) Changes in catch trends (Cav) for *B*. *vaillantii* against changes in the RCCI’ over the historical 34-year period (1972 to 2006).
(PDF)

S2 Fig The monthly length-frequency-distributions (LFDs) of *B*. *rousseauxii*. Length-frequency histograms with the growth curves (dashed lines) obtained through the bootstrapped ELEFAN with GA analysis for *B*. *rousseauxii* from (A) Madre de Dios River (2003–2005) and (B) the Ucayali-Urubamba River (2004–2005). The bars represent the restructured length frequency data, where black bars indicate positive peaks and white bars represent negative peaks, emphasized by the weak blue and red colors, respectively [131].
(PDF)

S3 Fig Length curve for the *B*. *rousseauxii* in the western Amazon region. Curve swarms (grey lines) and 95% confidence contours (dashed lines) for the *B*. *rousseauxii* in (A) Madre de Dios River and (B) Ucayali-Urubamba River. The thick black line is the growth curve representing the kernel density distribution mode (maximum density peak). Full Bootstrap, Nruns = 1000. The ELEFAN_GA fit algorithm was optimized for precision [131].
(PDF)

S4 Fig Linearized length-converted catch curve for *B*. *rousseauxii* captured in the western Amazon. (A) Madre de Dios River; (B) Ucayali-Urubamba River. Closed circles represent the data points used in the regression analysis to estimate Z = instantaneous rate of total mortality [132].
(PDF)

S5 Fig Yield per recruitment curve for *B*. *rousseauxii* caught in western Amazon region. (A) Madre de Dios River. (B) Ucayali-Urubamba River. F_0.5_ represents the fishing mortality at 50% of the biomass compared to the unexploited population, and F_MSY_ the fishing mortality at maximum sustainable yield.
(PDF)

S1 Table The annual catch of *B*. *vaillantii* (tons) by the trawler fishing fleet and the corresponding relative rate of catch increase (RRCI) and catch increase smoothed RRCI (RRCI’), the averaged previous catches (Cav), and the F/FMSY ratio for the period between 1972 and 2011. (XLSX)

S2 Table Annual maximum production of long-distance migratory species by states or departments for the period between 1980 and 2007. (XLSX)

S3 Table Annual maximum production of long-distance migratory species by city based on reliable or available data for the period between 1976 and 2019. (XLSX)

S1 Text (DOCX)
