# Supplementary figures and images for: Proactively averting the collapse of Amazon fisheries based on three migratory flagship species
Source: PLoS One. 2022 Mar 2;17(3):e0264490. doi: 10.1371/journal.pone.0264490 (PMC8890642; doi:10.1371/journal.pone.0264490)

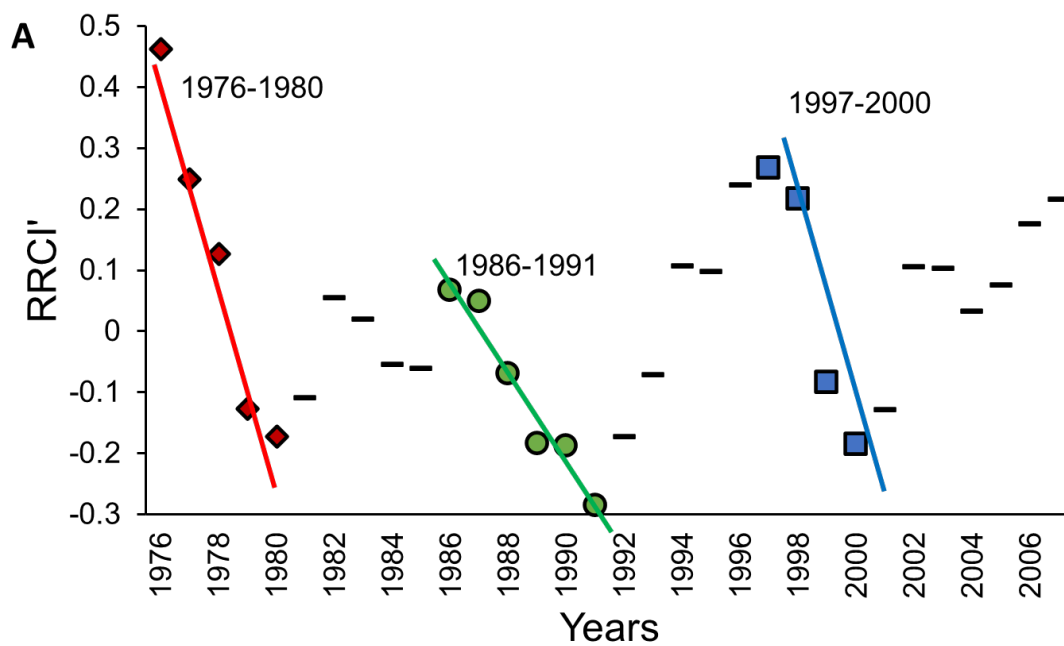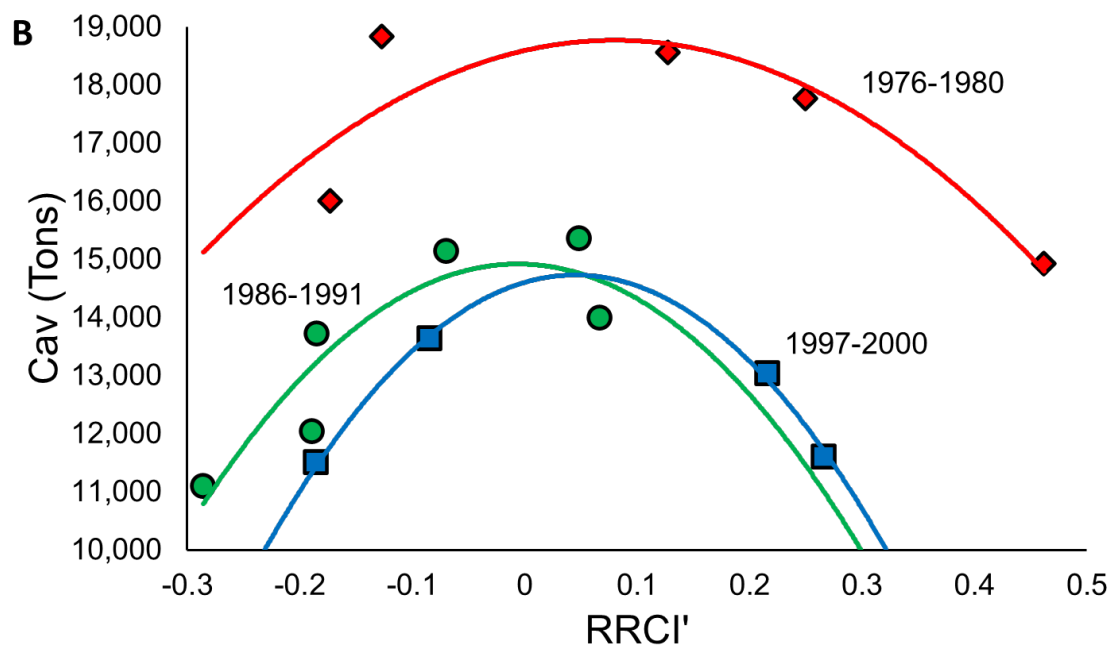

Supplement: S1 Fig — (A) Changes over time in the RRCI of B. vaillantii captured in the Amazon River estuary during a 34-year period (1972 to 2006). Years corresponding to significant changes in the fishery were not used to estimate the maximum yield and are represented by a black dash. Data with minor changes used for the regression are represented by diamonds, squares, and circles for each catch trend of years. (B) Changes in catch trends (Cav) for B. vaillantii against changes in the RCCI’ over the historical 34-year period (1972 to 2006). (PDF) [file pone.0264490.s001.pdf]

**A**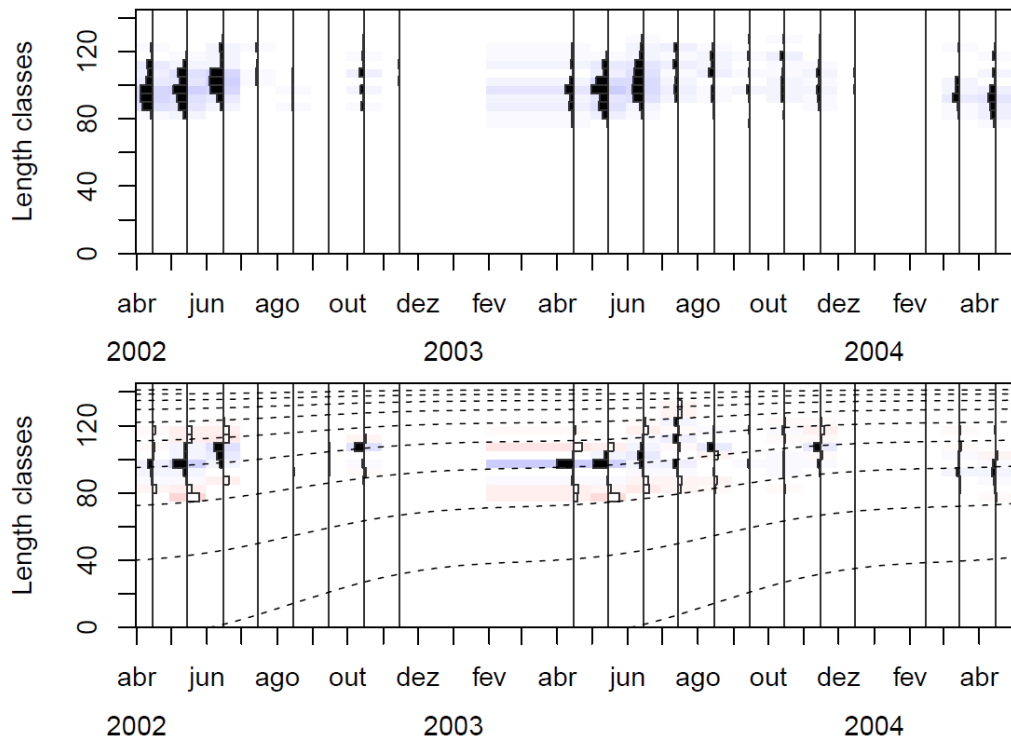**B**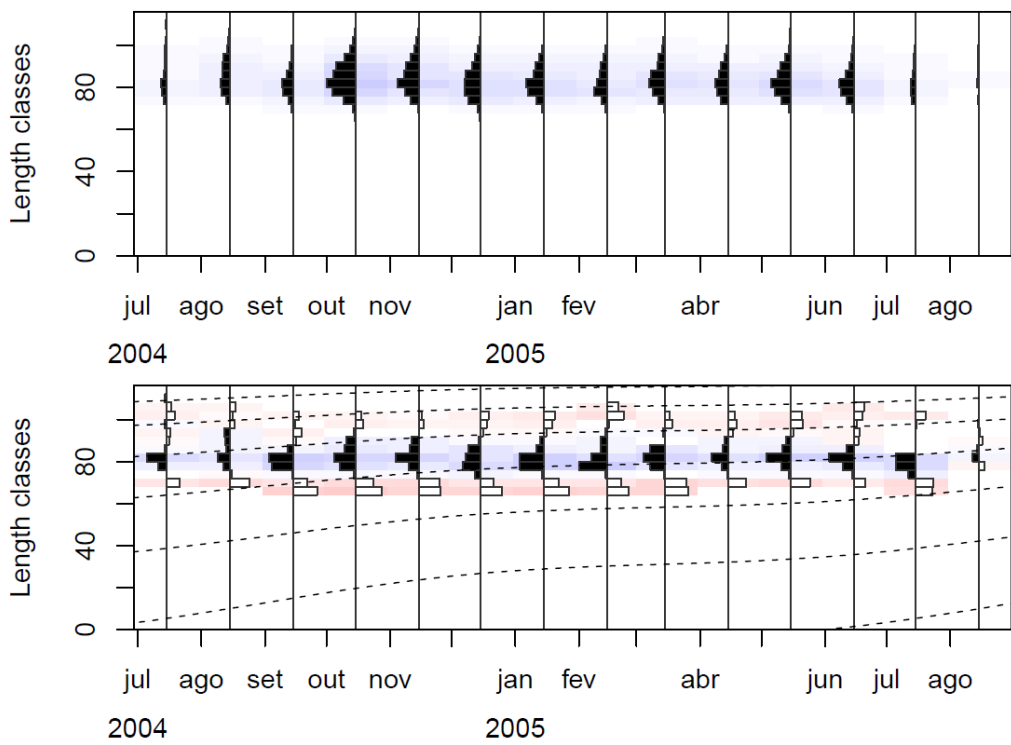

Supplement: S2 Fig — Length-frequency histograms with the growth curves (dashed lines) obtained through the bootstrapped ELEFAN with GA analysis for B. rousseauxii from (A) Madre de Dios River (2003–2005) and (B) the Ucayali-Urubamba River (2004–2005). The bars represent the restructured length frequency data, where black bars indicate positive peaks and white bars represent negative peaks, emphasized by the weak blue and red colors, respectively [131]. (PDF) [file pone.0264490.s002.pdf]

**A**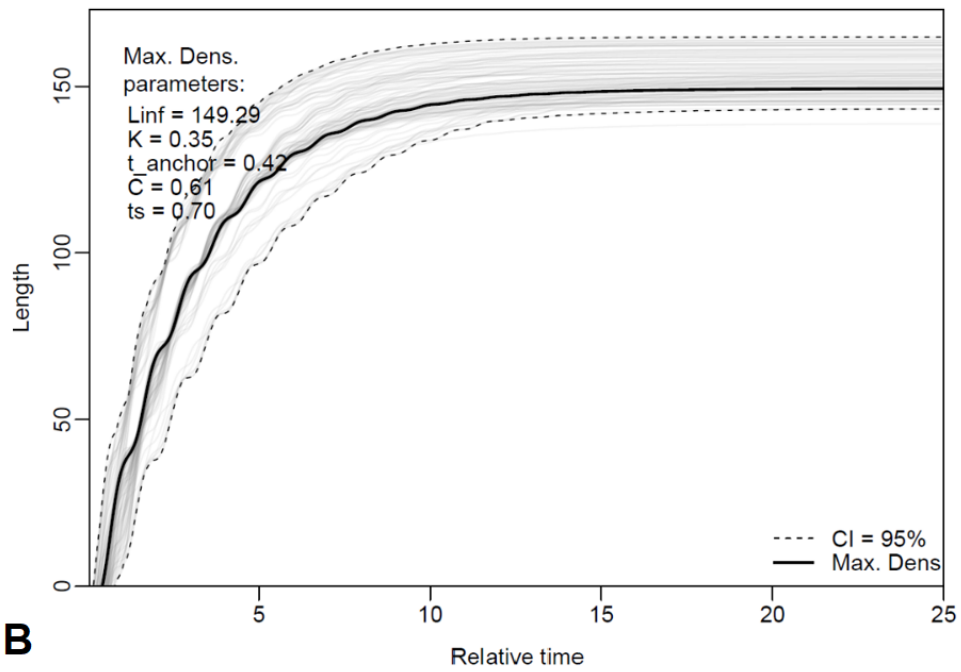**B**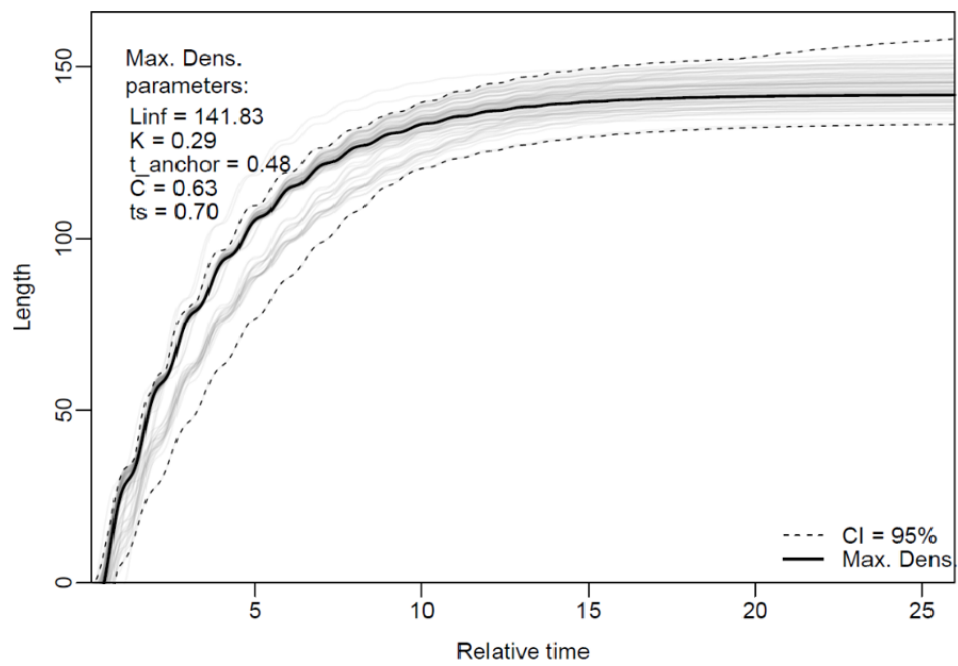

Supplement: S3 Fig — Curve swarms (grey lines) and 95% confidence contours (dashed lines) for the B. rousseauxii in (A) Madre de Dios River and (B) Ucayali-Urubamba River. The thick black line is the growth curve representing the kernel density distribution mode (maximum density peak). Full Bootstrap, Nruns = 1000. The ELEFAN_GA fit algorithm was optimized for precision [131]. (PDF) [file pone.0264490.s003.pdf]

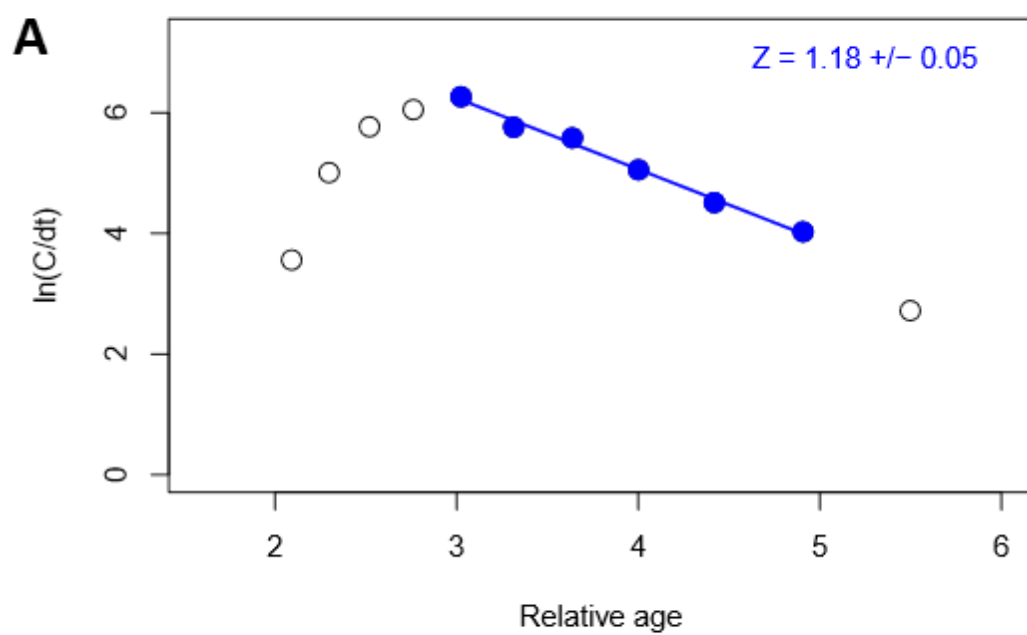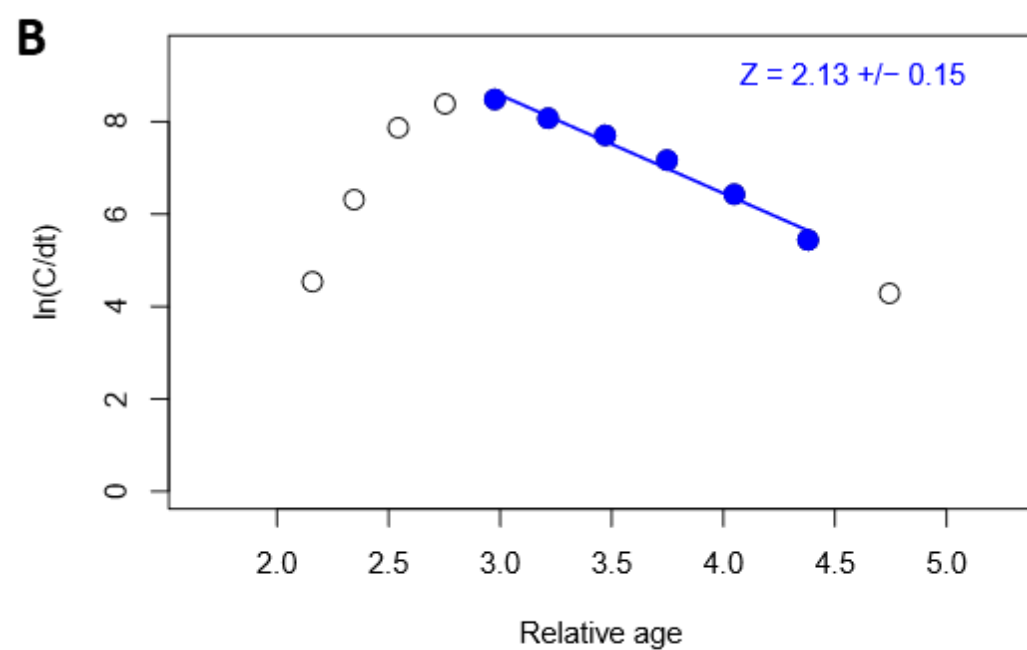

Supplement: S4 Fig — (A) Madre de Dios River; (B) Ucayali-Urubamba River. Closed circles represent the data points used in the regression analysis to estimate Z = instantaneous rate of total mortality [132]. (PDF) [file pone.0264490.s004.pdf]

**A**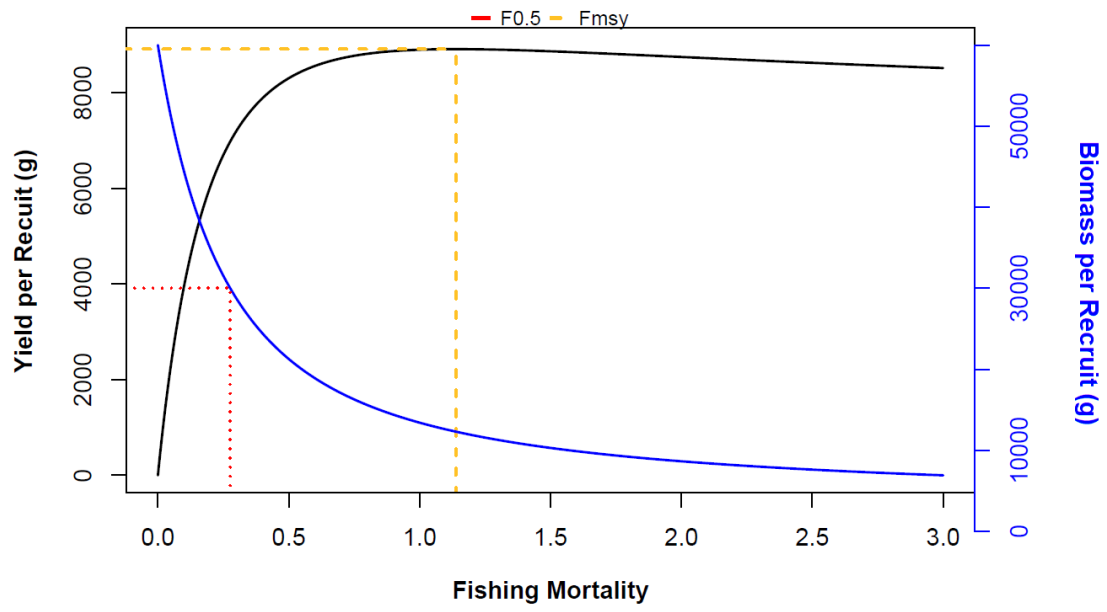**B**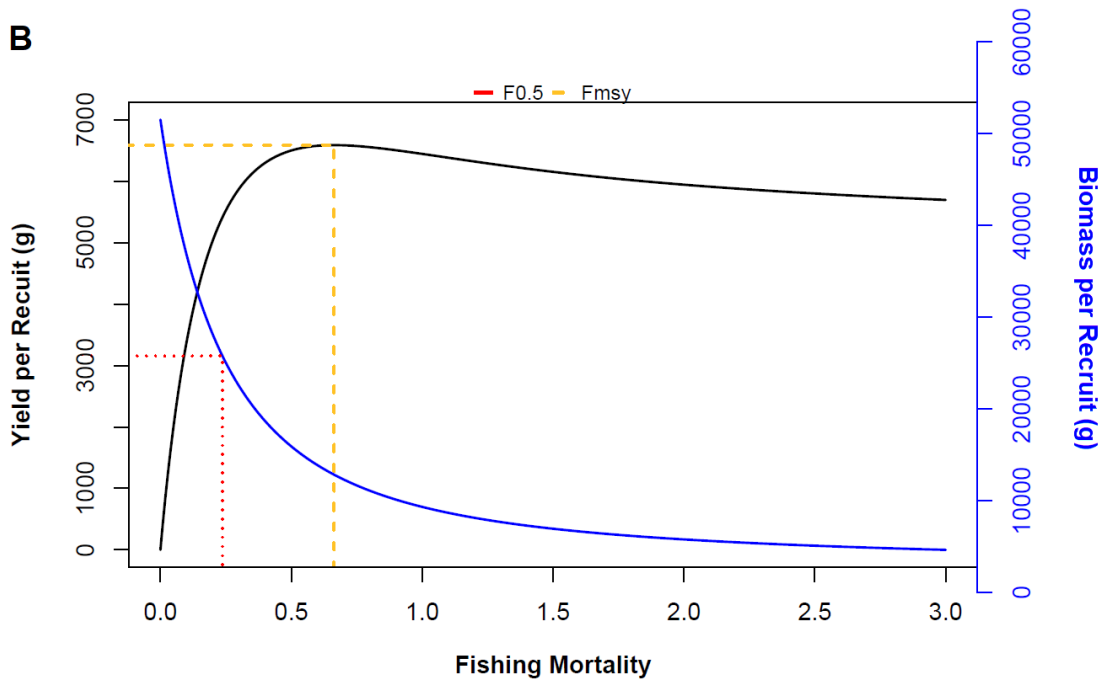

Supplement: S5 Fig — (A) Madre de Dios River. (B) Ucayali-Urubamba River. F0.5 represents the fishing mortality at 50% of the biomass compared to the unexploited population, and FMSY the fishing mortality at maximum sustainable yield. (PDF) [file pone.0264490.s005.pdf]
