# Supplementary material for: Proactively averting the collapse of Amazon fisheries based on three migratory flagship species
Source: PLoS One. 2022 Mar 2;17(3):e0264490. doi: 10.1371/journal.pone.0264490 (PMC8890642; doi:10.1371/journal.pone.0264490)
Supplement: S1 Text — (DOCX) [file pone.0264490.s009.docx]

# Supplementary material

# Methods

## Fisheries landing data

Fisheries landing data for goliath catfishes and *C. macropomum* in the Amazon were compiled from national fisheries agencies, scientific literature, government reports, and a fish landing database for Bolivia, Peru, Colombia, and Brazil. These data were organized by departments or states and by urban centers, which include public markets and refrigeration plants (Fig 1). The annual fisheries production statistics for Colombian departments and Brazilian states are from their national fisheries agencies and grouped by species, and in Brazil also by fishing fleet type (industrial or artisanal). Brazilian fisheries data include those from nine states (Acre, Amapá, Amazonas, Maranhão, Mato Grosso, Pará, Rondônia, Roraima and Tocantins) for the periods 1980-1989 and 1996-2007 that are available online from the Chico Mendes Institute for Biodiversity Conservation (ICMBIO n.d.). Colombian Amazonian fishery production includes ports in the Department of Amazonas, especially in the cities of Leticia and La Pedrera, and covers the periods 1993-1999 and 2006-2007, and are available online by the Colombian Fishery Agency “Servicio Estadístico Pesquero Colombiano” (SEPEC n.d.). Other than state or department data, urban center data are available for 43 cities in Brazil and three cities in Colombia and cover the period 1976- 2019, but no city has complete information for all years. The period covered by each city varied, ranging from one to 28 years. Peru has a long-time series of Amazonian fishery data where the Peruvian Fishing Agency (Dirección Regional de Pesqueria - DIREPRO) has collected data since 1980. Peruvian fisheries production data are available for the period 1980-1992 for the departments of Loreto and Ucayali (Barthem et al. 1995). Since 2000, Peruvian data are organized by 14 urban centers and available for Loreto, Ucayali, and Madre de Dios by DIREPRO. Fishery production for the Bolivian Amazon includes commercial fishing activity of 15 cities or towns for the years 1986-1994 of the departments of Pando, Beni, and Cochabamba (Van Damme et al. 2011) (Fig 1). Fishery production data from Brazilian urban centers in the period 1976-2019 were compiled from the literature (Goulding 1980, Isaac and Ruffino 2000, Doria et al. 2012a, Doria et al. 2012b); from refrigeration plant reports in Amazonas and Pará states produced by Brazilian Agency of Animal Production of the Agriculture Ministry “Ministério da Agricultura, Pecuária e Abastecimento” (MAPA); from the fish landing database of the Porto Velho Fishing Colony “Colônia de Pescadores Z-1, Tenente Santana” in Rondônia state; from many cities along the Amazon River in Amazonas and Pará states by IBAMA-Provárzea; and from many cities in Pará state by Eletronorte–Centrais Elétricas do Norte do Brasil, and Museu Paraense Emílio Goeldi (Fig 1). Ecuador's freshwater fishing activity is primarily for subsistence and local market supply, and minimal recorded data are available, not sufficient to infer annual production (21). Also, the fisheries production of the countries on the northern border of the Amazon Basin, Venezuela, Guyana, Suriname, and French Guiana, was not considered in the study due to the low fishing activity in this region.

## Fish length data

Goliath catfish lengths were compiled from the literature, technical reports, and commercial and experimental fishery monitoring programs at various periods between 1982 and 2011. *Brachyplatystoma vaillantii* length data were obtained from commercial and experimental fisheries in the Amazon River estuary in Brazil for 16 years between 1978 and 2011. *Brachyplatystoma rousseauxii* length data were collected from the commercial fisheries in Peruvian headwaters of the Ucayali-Urubamba (2004 to 2005) and Madre de Dios rivers (2002 to 2004) in Peru.

## Analyses of the status of fishery exploitation

Two packages implemented in R (R_Core_Team 2020) (‘TropFishR’) (Mildenberger et al. 2017) and ‘fishboot’ (Schwamborn et al. 2019) and the FiSAT II (Gayanilo et al. 2005) were utilized to estimate the population parameters of both goliath catfish species. The growth parameters (L∞ and K) and the confidence intervals (ICs 95%) of *B. rousseauxii* sampled in the headwaters of Ucayali River (2004-2005) and Madre de Dios River (2002-2004) were estimated by analyzing monthly length-frequency-distributions (LFDs) using the optimization algorithms associated with ELEFAN methods with non-parametric bootstrapping (Schwamborn et al. 2019). The growth parameters of *B. vaillantii* were estimated as L∞ = 110.5 cm and K =0.10 (Alonso and Pirker 2005). The mortality parameters were estimated by the following methods: total mortality (Z) by linearized length-converted catch curve method (LCC) (Pauly 1983), the natural mortality (M) by empirical method based on indirect estimators of M (Then et al. 2015), and the fishery mortality (F) by F = Z – M. The F_MSY_ of *B. rousseauxii* was estimated by the relation of yield per recruit of the Thompson and Bell model (Mildenberger et al. 2017) and for *B. vaillantii* it was considered as 0.30 year-1 (Alonso and Pirker 2005).

We estimated the maximum sustainable yield of *B. vaillantii* captured by the industrial fishery in the estuary by the relative rate of catch increase (RRCI) method (Equation 1) (Grainger and Garcia 1996, Gaertner et al. 2001):

${RRCI}_{t}=\frac{\left[ C_{t}-\left[ \frac{\left( C_{t-1}+C_{t-2}+C_{t-3} \right)}{3} \right] \right]}{\left[ \frac{\left( C_{t-1}+C_{t-2}+C_{t-3} \right)}{3} \right]}$ (Equation 1)

Where RRCI_t_= rate of catch increase in year t and C_t_ = catch in same year.

The period limits of the gradual development of the trawl fishery were determined by the set of years where the smoothed RRCI’ (Equation 2) exhibits a declining trend:

$\mathrm{RRCI}_{t}^{'}=\left( \frac{\sum_{t-1}^{t+1} {RRCI}_{t}}{3} \right)$ (Equation 2)

Where RRCI’_t_= smoothed RRCI_t_.

The principle of the RRCI’_t_ is that when it declines to zero the fishery reaches the mature phase, and the catch reaches the maximum sustainable yield, with the model assumption that the fishing effort had increased over time (Grainger and Garcia 1996, Gaertner et al. 2001). This assumption is consistent with the trawl fishing effort for *B. vaillantii* which increased over time despite a regulation in 2002 prohibiting more than three boats fishing together (Barthem et al. 2015). The mean life expectancy of an individual in the fishable population (L), used to calculate the averaged previous catches (Cav) (Equation 3), was considered as the age groups that are vulnerable to fisheries in the estuary, which represents the first three years of life before the goliath catfishes migrate. The second-degree polynomial was used to fit the regression between RRCI’ and Cav, to estimate the maximum sustainable yield and the confidence intervals for each set of years:

${Cav}_{t}=\left( \frac{1}{L} \right)\sum_{i=0}^{L} C_{t-i}$ (Equation 3)

Where Cav_t_= averaged previous catches of year t and L = the mean life expectancy of an individual in the fishable population

The MSY, F_MSY,_ and F/F_MSY_ reference points derived from these models indicated the stock status of both species in each Amazon region. The *B. vaillantii* stock in the estuary was considered overfished if the catch exceeded the approximate MSY (24), or if the indices F/ F_MSY_ were larger than one, otherwise it was considered “healthy” for all stocks and regions (Pons et al. 2017).

# References

Alonso, J. C., and L. E. M. Pirker. 2005. Dinâmica populacional e estado atual da exploração de piramutaba e de dourada. Pages 21-28 *in* N. N. Fabré and R. B. Barthem, editors. O Manejo da Pesca dos Grandes Bagres Migradores. Instituto Brasileiro do Meio Ambiente e dos Recursos Naturais Renováveis, Brasília.

Barthem, R. B., H. Guerra, and M. Valderrama. 1995. Diagnostico de los recursos hidrobiológicos de la Amazonía. Tratado de Cooperación Amazonica, Lima, Peru.

Barthem, R. B., A. Mello-Filho, W. Assunção, and P. F. F. Gomes. 2015. Estrutura de tamanho e distribuição espacial da piramutaba (*Brachyplatystoma vaillantii*) na foz Amazônica: implicações para o manejo da pesca. Bol. Inst. Pesca, São Paulo **41**:249-260.

Doria, C. R. d. C., M. A. L. Lima, A. R. d. Santos, S. T. B. d. Sousa, A. L. B. Mojica, S. A. d. S. Monteiro, and M. Petrere-Jr. 2012a. Subprograma De Monitoramento Da Atividade Pesqueira. Energia Sustentável do Brasil, Porto Velho- RO.

Doria, C. R. d. C., M. L. Ruffino, N. C. Hijazi, and R. L. d. Cruz. 2012b. A pesca comercial na bacia do rio Madeira no Estado de Rondônia, Amazônia brasileira. Acta Amaz. **42**:29-40.

Gaertner, D., A. Fonteneaub, and F. Laloëa. 2001. Approximate estimate of the maximum sustainable yield from catch data without detailed effort information: Application to tuna fisheries. Aquat Living Resour **14** 1−9.

Gayanilo, F. C., P. Sparre, and D. Pauly. 2005. FAO-ICLARM Stock Assessment Tools II (FiSAT II)—Revised version. FAO, Rome.

Goulding, M. 1980. The Fishes And The Forest : Explorations in Amazonian Natural History. University of California Press, Berkeley & Los Angeles.

Grainger, R. J. R., and S. M. Garcia. 1996. Chronicles of marine fishery landings (1950-1994): trend analysis and fisheries potential. FAO, Roma.

ICMBIO. n.d. Estatística por região.

Isaac, V. J., and M. L. Ruffino. 2000. A estatística pesqueira no Baixo Amazonas: Experiencia do Projeto Iara. IBAMA. Coleção Meio Ambiente. Série Estudos Pesca **22**:201-224.

Mildenberger, T. K., M. H. Taylor, M. Wolff, and S. Price. 2017. TropFishR: an R package for fisheries analysis with length‐frequency data. Methods Ecol. Evol **8**:1520-1527.

Pauly, D. 1983. Length-converted catch curves: a powerful tool for fisheries research in the tropics (part 1). Fishbyte **1**:9-13.

Pons, M., T. A. Branch, M. C. Melnychuk, O. P. Jensen, J. Brodziak, J. M. Fromentin, S. J. Harley, A. C. Haynie, L. T. Kell, M. N. Maunder, A. M. Parma, V. R. Restrepo, R. Sharma, R. Ahrens, and R. Hilborn. 2017. Effects of biological, economic and management factors on tuna and billfish stock status. Fish Fish **18**:1-21.

Schwamborn, R., T. K. Mildenberger, and M. H. Taylor. 2019. Assessing sources of uncertainty in length-based estimates of body growth in populations of fishes and macroinvertebrates with bootstrapped ELEFAN. Ecol Model **393**:37-51.

SEPEC. n.d. Boletines estadísticos pesqueoros y de la acuicultura.

Then, A. Y., J. M. Hoenig, N. G. Hall, and D. A. Hewitt. 2015. Evaluating the predictive performance of empirical estimators of natural mortality rate using information on over 200 fish species. ICES J Mar Sci **72**:82-92.

Van Damme, P. A., F. M. Carvajal-Vallejos, A. Rua, L. Córdova, and P. Becerra. 2011. Pesca comercial en la cuenca amazónica boliviana. Pages 247-291 *in* P. A. Van Damme, F. M. Carvajal-Vallejos, and J. Molina Carpio, editors. Los peces y delfines de la Amazonía boliviana: hábitats, potencialidades y amenazas. INIA, Cochabamba, Bolivia.
